# PROTAC-based protein degradation: a window of opportunity for melanoma therapy

**DOI:** 10.1186/s12929-026-01225-2

**Published:** 2026-02-27

**Authors:** Giulia Gentile, Simona D’Aguanno, Marta Di Martile, Adele Petricca, Elisabetta Valentini, Stefano Scalera, Donatella Del Bufalo

**Affiliations:** 1 Preclinical Models and New Therapeutic Agents Unit, IRCCS Regina Elena National Cancer Institute, Via Elio Chianesi 53, 00144 Rome, Italy; 2 Biostatistics, Bioinformatics and Clinical Trial Center, IRCCS Regina Elena National Cancer Institute, Via Elio Chianesi 53, 00144 Rome, Italy

**Keywords:** Melanoma, Targeted protein degradation, Proteolysis targeting chimera, Preclinical studies

## Abstract

The key small molecule-based modalities for inducing targeted protein degradation have seen explosive growth over the past decade. They include heterobifunctional degraders such as PROteolysis TArgeting Chimeras (PROTACs): molecules working as protein degraders by inducing proximity between a protein of interest, mostly a disease-causing protein, and an ubiquitin E3 ligase to trigger protein ubiquitination and degradation. The power of PROTACs has been broadly demonstrated, and their success has motivated interest and efforts in expanding the concept to several diseases including cancer, in the hope to tackle previously elusive or inadequately drugged targets and accelerate translation to clinical therapies. Some PROTACs have advanced to clinical development, confirming the efficacy and feasibility of this innovative therapeutic approach*.* Today, over 40 degraders, including PROTACs, are being developed in clinical trials, many for oncology indications. Although the literature is particularly abundant in reviews on PROTACs, there are currently no studies that collect data on the use of PROTACs in cutaneous melanoma, the most common and aggressive type of skin cancer. Therefore, in this comprehensive review, preclinical findings will be presented and discussed, helping to bring together studies and efforts in the rapidly evolving field of PROTACs, with regard to cutaneous melanoma. Thus, offering an opportunity for scientists and clinicians to deepen their knowledge about this field, and to shape the future of personalized cancer therapy.

## Introduction

**Fig. 1:**
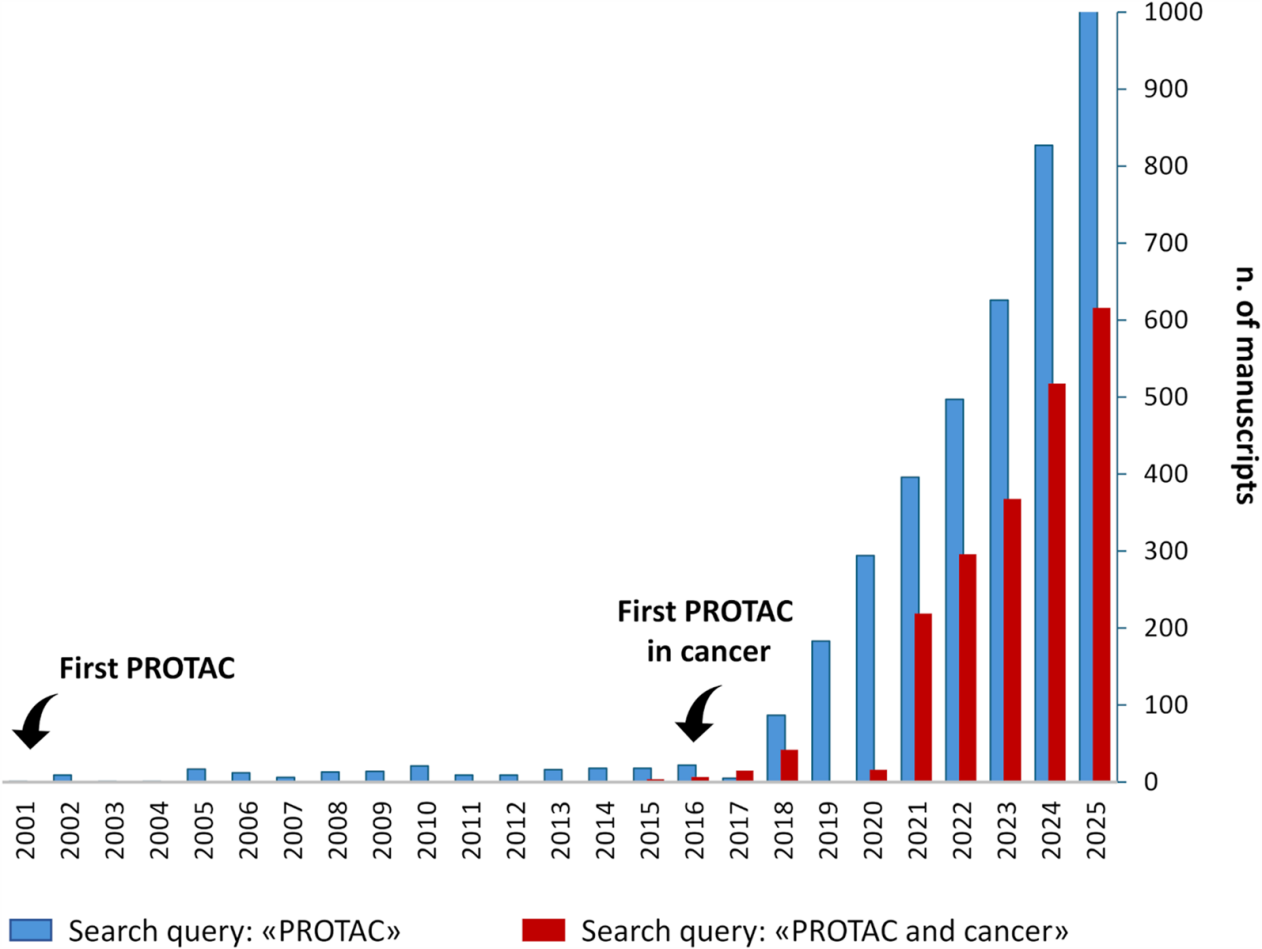
Preclinical studies, clinical trials and reviews in pubmed [94]. Search queries: “PROTAC” (blue bars) and “PROTAC and cancer” (red bars). Manuscripts published from January 2001 to December 2025 have been considered

Initially described in the early 2000s, PROteolysis TArgeting Chimeras, PROTACs, have revolutionized drug discovery and therapy of different diseases, including cancer [1, 2]*.* Over the last 25 years, an increasing number of studies have been conducted on the use of PROTACs in tumor models of different origins (about 65% of total studies), including cutaneous melanoma (about 2% of total studies on cancer) (Fig. 1) with some PROTACs entering clinical trials [3] (Table 1**)**.

**Table 1:** PROTACs in clinical trials for malignant diseases

Target protein (POI)	PROTAC	E3 ligase	Trial number***	Condition*	Treatment	Phase	Status	Results
Androgen Receptor (AR)	ARV-110	CRBN	NCT03888612	Castration resistant prostate cancer	ARV-110	I/II	Completed	N/A
			NCT05177042	Castration resistant prostate cancer	ARV-110 + Abiraterone	I	Completed	N/A
	ARV-766	VHL	NCT05067140	Prostate cancer	ARV-766 ARV-766 + Abiraterone	I/II	Active	N/A
	BMS-986365 (CC-94676)	VHL	NCT04428788	Castration resistant prostate cancer	BMS-986365	I	Active	N/A
			NCT06417229	Healthy adult males	BMS-986365 BMS-986365 + Rabeprazole	I	Completed	N/A
			NCT06433505	Healthy adult males	BMS-986365	I	Completed	N/A
			NCT07242781	Healthy adult males	BMS-986365 BMS-986365 + Itraconazole BMS-986365 + Rifampin	I	Not yet recruiting	N/A
			NCT06764485	Castration resistant prostate cancer	BMS-986365 Enzalutamide Abiraterone Docetaxel Predinsone/Prednisolone	III	Recruiting	N/A
Bcl-xL	DT2216	VHL	NCT04886622	Solid tumors** Hematologic malignancy	DT2216	I	Completed	N/A
			NCT06620302	Solid tumors** Fibrolamellar carcinoma	DT2216 + Irinotecan	I/II	Recruiting	N/A
			NCT06964009	Ovarian Cancer	DT2216 + Paclitaxel	I	Recruiting	N/A
BRAF-V600E	CFT1946	CRBN	NCT05668585	BRAF-V600mut melanoma, Non-small cell lung cancer, Colon-rectal cancer, Anaplastic thyroid cancer and other solid Cancers**	CFT1946 CFT1946 + Trametinib CFT1946 + Cetuximab	I	Completed	N/A
Bromodomain-containing Protein 9 (BRD9)	RNK05047	HSP90	NCT05487170	Advanced solid cancers** Diffuse large B-cell lymphoma	RNK05047	I/II	Recruiting	N/A
Bromodomain-containing Protein 9 (BRD9)	CFT8634	CRBN	NCT05355753	Synovial sarcoma SMARCB1-null solid cancer SMARCB1-perturbed cancer	CFT8634	I/II	Terminated	N/A
	FHD609	CRBN	NCT04965753	Advanced Synovial Sarcoma	FHD609	I	Terminated	N/A
Bruton's Tyrosine Kinase (BTK)	NX-5948	CRBN	NCT05131022	Chronic lymphocytic leukemia Small lymphocytic lymphoma Diffuse large B cell lymphoma Follicular lymphoma Mantle cell lymphoma Marginal zone lymphoma Primary central nervous system lymphoma	NX-5948	I	Recruiting	N/A
			NCT06717269	Healthy adults	NX-5948 NX-5948 + Esomeprazole	I	Recruiting	N/A
			NCT06691828	Healthy adults	NX-5948	I	Completed	N/A
			NCT06593457	Healthy adults	NX-5948	I	Completed	N/A
			NCT07221500	Chronic lymphocytic leukemia Small lymphocytic lymphoma	NX-5948	II	Recruiting	N/A
	NX-2127	VHL	NCT04830137	Chronic lymphocytic leukemia Small lymphocytic lymphoma Waldenstrom macroglobulinemia Mantle cell lymphoma Marginal ZONE LYMPHOMa Follicular lymphoma Diffuse large B-cell lymphoma Primary central nervous system lymphoma	NX-2127	I	Recruiting	N/A
Estrogen Receptor (ER)	ARV-471	CRBN	NCT05930925	Healthy adults	ARV-471	I	Completed	N/A
			NCT04072952	Breast Cancer (ER + /HER2−)	ARV-471 + Palbociclib	I/II	Active	N/A
			NCT06206837	Breast Cancer (ER + /HER2−)	ARV-471 + PF-07220060	I/II	Active	N/A
			NCT05548127	Breast Cancer (ER + /HER2−)	ARV-471 + Abemaciclib	I/II	Active	N/A
			NCT05654623	Breast Cancer (ER + /HER2−)	ARV-471 vs Fulvestrant	III	Active	N/A
			NCT05573555	Breast Cancer (ER + /HER2−)	ARV-471 + Ribociclib	I/II	Active	N/A
			NCT05501769	Breast Cancer (ER + /HER2−)	ARV-471 + Everolimus	I	Completed	N/A
			NCT06125522	Breast Cancer (ER + /HER2−)	ARV-471 + Samuraciclib	I/II	Active	N/A
			NCT05732428	Breast Cancer (ER + /HER2−)	ARV-471	I	Completed	N/A
			NCT05538312	Healthy adults	ARV-471 ARV-471 + Itraconazole	I	Completed	A
			NCT05673889	Healthy adults	ARV-471 + Dabigatran etexilate	I	Completed	A
			NCT05652660	Healthy adults	ARV-471 + Rosuvastatin	I	Completed	A

^*^Patients enrolled for the listed clinical trials have advanced or metastatic cancers and had previously undergone first-line therapy without success, except for healthy patients. **Studies performed on solid cancers including melanoma. ***https://clinicaltrials.gov/. *N/A* Not Available, *A* Available

**Fig. 2:**
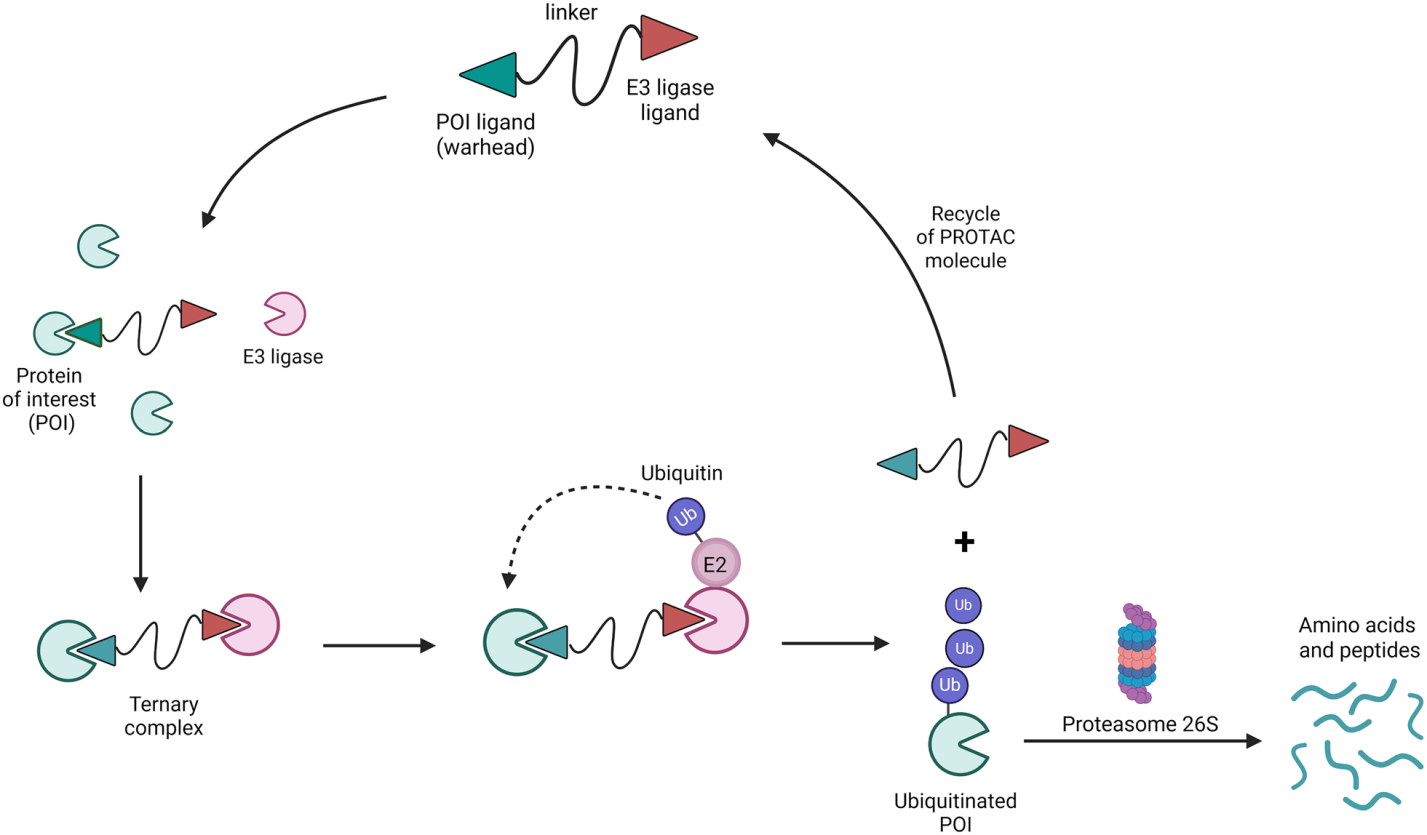
Schematic representation of PROTAC. Created by BioRender

PROTACs represent a new strategy to treat diseases through degradation of a Protein Of Interest (POI), as well as a new tool for chemical biology studies or for in-depth interrogation of specific protein biology [4–7]*.* They are heterobifunctional molecules composed by a linker connecting a targeting ligand (warhead) for a POI with an E3 ligase for polyubiquitination and consequent proteasome degradation of the POI (Fig. 2).

Shortening the distance between the POI and the E3 ligase, PROTACs belong to a large family of proximity-induced approaches which also includes protein blockers and stabilizers, protein post-translational modifiers, as well as agents for cell therapy [8]. Unfortunately, not all ternary complexes, POI–PROTAC–E3 ligase, are effective in inducing degradation: in some cases, unfavorable ternary complexes are evidenced due to the Hook effect, a phenomenon in which high PROTAC concentrations decrease its activity due to the formation of inactive binary complexes. Thus, unexpected decrease in POI degradation can be observed despite high concentration of PROTACs [9]. On the other hand, some PROTACs showing weak binding affinity for the target protein have been reported to induce strong protein degradation [6, 10, 11].

Although the possibility to target cell surface proteins and receptors, as well as proteins involved in protein–protein interactions, by PROTACs is limited, the advantage is that they can lead to the degradation of proteins defined as undruggable/unreachable with other currently available approaches, such as transcription factors [12], scaffolding proteins [13] or RNA-binding proteins [14, 15]. When compared to small-molecule inhibitors, macromolecular antibodies or small interfering RNA, PROTACs also present other advantages. These include the ability to eliminate the target proteins, efficacy at low doses, reduced toxic side-effects, increased selectivity, higher potency, and longer pharmacological effects due to their recycling nature.

Integrating data from different publicly available data sources and taking into consideration deep learning/artificial intelligence-aided drug design, protein characteristics and available ligands, several studies investigated PROTAC’s development leading to lists of potential proteins susceptible to PROTACs. Consequently, terms as PROTACable, target PROTACability or PROTACpedia have been created [16–18]. Human proteome analysis led to the PROTACtable genome list and to the identification of more than one thousand PROTACtable proteins, whose degradation could be induced using PROTACs, but for which PROTACs are not yet available [17]. These approaches also led to the identification of protein targets for which there are no drugs currently approved in clinical practice or under study in clinical trials. Due to the intranuclear or intracellular distribution, or to the relatively smooth surface, about 85% of proteins related to specific disease miss targeted drugs [15]. Considering both experimental findings and structural information, a web-based database freely accessible, PROTAC-DB, lead to the identification of about 6000 PROTACs, 2700 linkers, 100 E3 ligands and 570 warheads. This database provided information in terms of PROTAC’s binding affinity, degradation, crystal structure, pharmacokinetic parameters and cellular activities [19, 20].

While a restricted number of PROTACs reached the phase I-III clinical trial stage for oncological disease and showed promising results in terms of safety and pharmaceutical behavior (Table 1: PROTACs in clinical trials for malignant diseases), most of them have only been tested in preclinical cancer models*.*

**Fig. 3:**
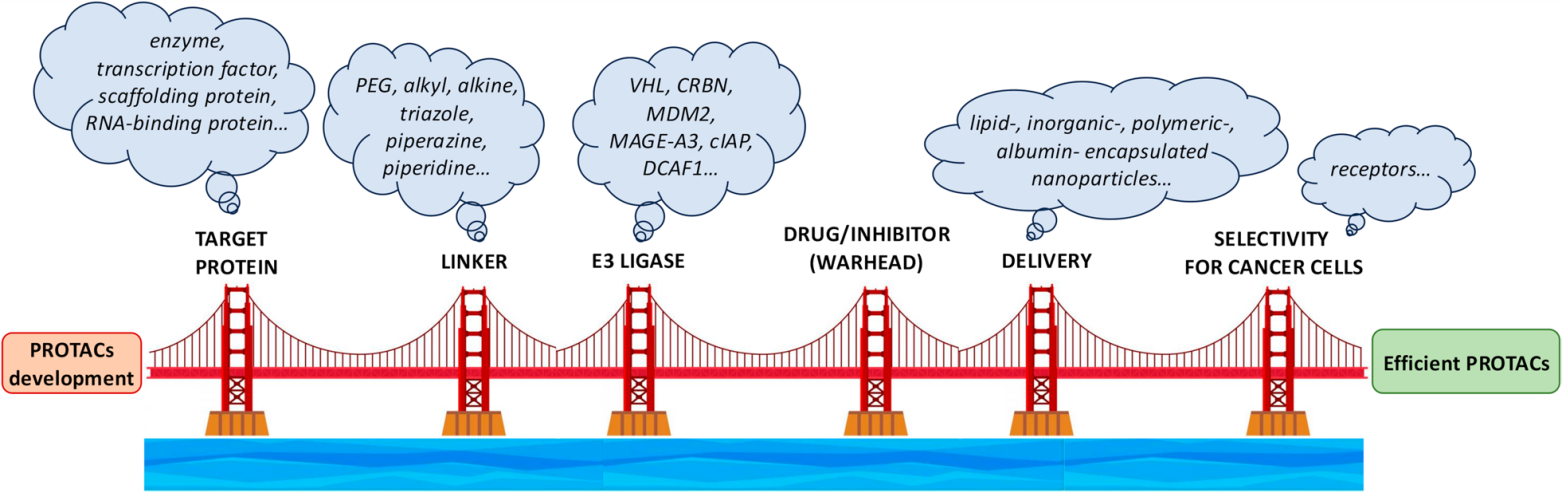
PROTACS as a bridge for cancer therapy. Each pillar represents a possible factor relevant to optimize PROTACs design for the treatment of melanoma or other tumor histotypes or, more in general, malignant diseases. Created by PowerPoint

There are several variables that influence the efficacy of a PROTAC such as E3 ligase [5, 21–29], ligand connecting the protein of interest with an E3 ligase [30, 31], and the drug used to develop the PROTAC [32] (Fig. 3).

The Von Hippel-Lindau (VHL) and Cereblon (CRBN) proteins, are the most used E3 ligase but present the limit of widespread expression or resistance for their inactivity [33]. Among the about 600 E3 ligases contained in the human genome, approximately 40% plays a role in the Ubiquitin–Proteasome System (UPS) and only about 2% have been identified as a valid strategy to design PROTACs [5, 21–29, 34].

Influencing the binding between the various components of the PROTAC or the conformation, orientation, mobility, stability and physicochemical properties of the complex, also linker type/composition and length represent a field of study in the design and synthesis of efficient PROTACs. The use of “photoswitchable” and “clickable” linkers, as well as rigidifying motifs, was taken a step further by several researchers [30, 31]*.* Very recently, single amino acid-based/linker-free PROTACs have been designed and reported to induce protein degradation and in vitro/in vivo antitumor activity [35].

**Fig. 4:**
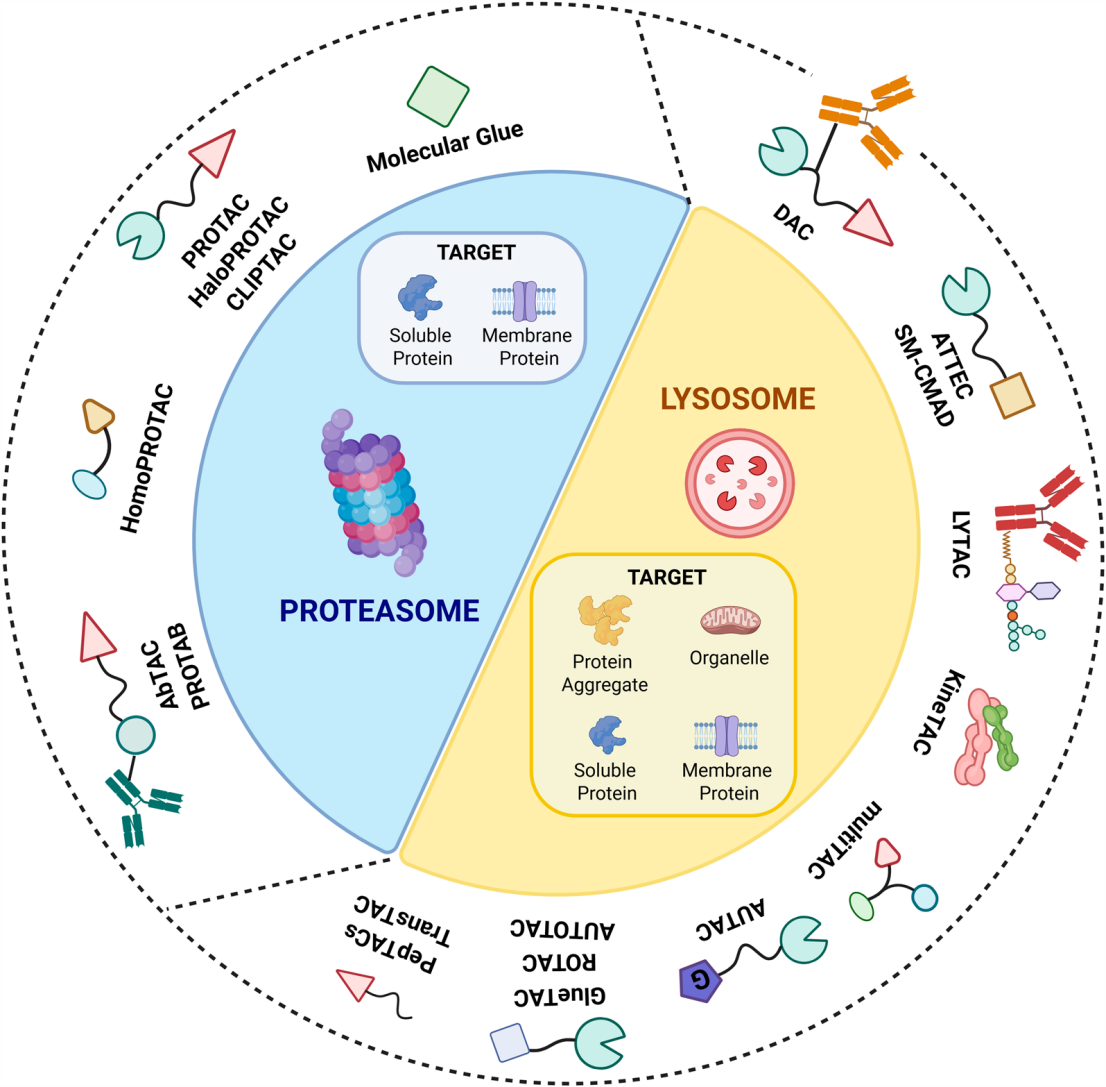
Targeted protein degraders acting through Proteasome or Lysosome pathways. Created by BioRender

To increase PROTACs efficacy, especially against challenging target proteins, and the selectivity between normal and cancer cells, in the last years the concept of chemical, ultrasound, hypoxia or light controllable protein degradation has emerged (PHOtochemically TArgeting Chimeras [PHOTACs], opto-PROTACs, photo-PROTACs, PhotoDegradation-TArgeting Chimeras [PDTACs], smart-PROTACs, in-cell CLIck-formed Proteolysis TArgeting Chimeras [CLIPTACs], covalent PROTACs). Spatiotemporal control of PROTACs efficacy has been also developed [36–48]. HaloPROTACs recruiting E3 ubiquitin ligases on one side, and binding to a HaloTag fusion protein that is engineered to bind specifically to the POI on the other side, have been also designed. They have been employed as a proof-of-concept system and as a chemical biology tool to validate the feasibility of Targeted Protein Degradation (TPD) [49] (Fig. 4).

## Pathways for protein degradation beyond PROTACs

In addition to PROTACs, in the last years other TPD technologies (TACnologies) based on the proximity-induced modality have been developed using different systems [50–52]. Some of these strategies, overcome the limitation of transmembrane proteins degradation exploiting the capabilities of antibody targeting and internalization of cell surface proteins (Fig. 4). Several studies reported on the efficacy of these approaches in melanoma models.

TPD approaches include degraders engaging:

- The ubiquitin–proteasome system. Molecular Glues (MGs) consists of small molecules that can induce, facilitate or increase the binding between an E3 ligase and a POI with consequent polyubiquitination and degradation of the latter. MG is considered an effective protein degradation approach with a mechanism superimposable to that of PROTACs [53]*.* Rapamycin, Cyclosporin A and FK506 have been the first MGs discovered in the early 1990s [54]*.* In 2023, the pharmacological inhibitor of CDK12, SR-4835, has been reported to act as a MG able to induce cytotoxicity in the serine/threonine-protein kinase B-Raf (BRAF) mutated melanoma models through degradation of cyclin K [55, 56].
- The endosome-lysosome system. LYsosome TArgeting Chimeras (LYTACs) are heterobifunctional molecules comprising a recruiting molecule (e.g. an antibody) for a target protein joined by a linker to a ligand for receptors exposed in the cell membrane of the targeted cell/tissue. Lysosome-targeting receptors include Mannose 6-Phosphate Receptor (M6PR), Folate Receptor 1 (FOLR1), Epidermal Growth Factor Receptor (EGFR) and TransFerrin ReCeptor (TFRC/TfR). The ternary complex formed by the POI, the linker and the receptor, triggers internalization of the target protein through endocytosis and its subsequent lysosomal degradation. Although most LYTACs employ antibodies for the binding to the POI, they can also use specific peptides to destroy the POI (i.e. integrin-binding peptide, peptide-based LYTACs). Degrading also membrane proteins and extracellular soluble factors, LYTACs overcome the limitation of PROTACs, only degrading intracellular proteins [57]. Two recently published papers reported on the possibility to induce in vitro and in vivo antitumoral activity in melanoma models through degradation of Programmed -Death Ligand 1 (PD-L1) by LYTACs [58, 59]. Another strategy for endocytosis and lysosomal delivery is represented by cytoKine receptor-TArgeting Chimeras (KineTACs). KineTACs are bispecific antibodies formed by a cytokine arm binding its cognate receptor, and a POI-binding arm. They are able to degrade extracellular and cell surface proteins, such as PD-L1, Human Epidermal growth factor Receptor 2 (HER2), EGFR [60]*.* Unfortunately, there is no data available at the moment regarding the use of KineTAC in melanoma models.
  Multivalent TArgeting Chimera (MultiTAC) also employs lysosome degradation but, differently from LYTACs and KineTACs, it does not require lysosome-targeting ligands or membrane receptors. This technology has been designed for tumor-specific PD-L1 degradation thanks to the enzymatic and acidic behaviour of Tumor MicroEnvironment (TME). It integrates a PD-L1 ligand with a hydrophilic methexyl-PolyEthylene Glycol (PEG) segment cleavable by metalloproteinase-2 and a hydrophobic segment ionizable by pH acid.
  Nanobody-based GlueTAC has also been designed to direct membrane proteins or surface antigens, such as PD-L1, to lysosomal degradation via the conjugation of a cell-penetrating peptide with a lysosomal-sorting sequence [61]. In vitro and in vivo antitumor activity of tumor selective MultiTAC and GlueTAC degrading PD-L1 protein has been reported in mouse melanoma models [61, 62]*.*
  In 2023, scientists from the German cancer research center developed ROTACs, bispecific R-spondin chimeras that use domains from secreted R-spondin proteins to engage the membrane-localized E3 ligases ZNRF3/RNF43 for targeted lysosomal degradation of transmembrane proteins. PD-L1 protein degradation and inhibition of in vivo melanoma growth was induced by the R2PD1 ROTAC [63]*.*
  The Degrader Antibody Conjugates (DAC) also uses endocytosis mediated receptor to be internalized into the cell, with consequent fusion with endosomes, and transportation into lysosomes. Several proteins, such as BRD4 (BRomoDomain-containingprotein4), BRM/SMARCA2, G1 to S Phase Transition 1 (GSPT1), have been reported to be degraded by DAC with consequent induction of antitumor activity [64]. Of note, ORM-5029 is a first-in-class drug candidate designed to selectively deliver catalytic GSPT1 protein degraders to HER2-expressing advanced tumors via antibody targeting (NCT05511844; [3])*.* Although currently there are no data available regarding the use of DACs in melanoma models, we hope this approach will be extended to this tumor histotype, as reported for other cancer types [65].
  A stable-peptide based platform (PepTACs) through lysosomal targeted endocytosis has recently been developed for the degradation of POI by recycling transferrin receptor , which is frequently overexpressed in cancer cells [66, 67]*.* The TRANSferrin receptor TArgeting Chimera (TransTAC) also employs TFRC and the lysosomal degradation pathway after internalization of membrane proteins (PD-L1, EGFR, chimeric antigen receptor and cluster of differentiation 20 (CD))*.* This heterobispecific antibody modality demonstrated its efficacy in cancer cells not including melanoma [68].

- The autophagy-lysosome system. AUtophagy-TArgeting Chimera (AUTAC) (also known as AUTOTAC) and AuTophagosome TEthering Compounds (ATTEC) are heterobifunctional molecules using the autophagic mechanism to target intracellular proteins or organelles. AUTAC/AUTOTAC consist of a small unit (SQSTM1/p62 autophagosome receptor, LC3 key autophagy protein) binding the substrate and a degradation tag (generally an E3 ligase) connected by a flexible linker [69]*.* ATTEC are composed of a domain recruiting LC3 and a domain joining lipid particles [70]*.* Although the antitumor efficacy of AUTAC or ATTEC has not yet been demonstrated in preclinical models of melanoma, we hope that data will be generated in this direction as demonstrated in several preclinical cancer models [71].
  Also Split-and-Mix Chaperone-Mediated Autophagy-based Degraders (SM-CMAD) belong to this group of TPD: this technology demonstrated to knock down a range of proteins including Mitogen-activated protein Kinases 1 and 2 (MEK1/2) [72]. Compared to other lysosomal-based degraders, SM-CMAD approach offered the advantage of allowing an easy screening (as it lacks linker optimization) but, unfortunately, shows the disadvantage of high term maximal effective concentration. The efficacy of a SM-CMAD (KFERQ+Cobi-θθ) in degrading MEK1 and MEK2 has been evidenced in melanoma cells [72].

## Cutaneous melanoma features and treatment

Skin cancer is divided into 3 groups: squamous cell carcinoma, basal cell carcinoma, and cutaneous melanoma originating, from squamous cells, basal cells and melanocytes, respectively. Although it represents about 1% of all malignant skin tumors, cutaneous melanoma stands as the most aggressive and deadly form, causing about 2% of all cancer deaths in the world. Patients’ survival is closely related to the stage of the tumor and to the ability to make a timely diagnosis. More rarely, melanoma can also develop in extracutaneous sites and, depending on the site of origin, it may be of mucosal (mucosa) or uveal (uvea) type. Cutaneous melanoma, (hereafter melanoma), is one of the most highly mutated malignancies. High Tumor Mutational Burden (TMB) increases the neoantigens recognizable by T-cells, generally boosting immunotherapy response. However, high TMB could also generate phenotypic heterogeneity and clonal evolution, with subpopulations of cells with different proliferative/invasive state or expressing different level of Melanocyte Inducing Transcription Factor (MITF), finally causing resistance to therapy. The most altered signal transmission pathway in melanoma is that of RAS/RAF/MEK/ERK kinases (Mitogen Activated Protein Kinase, MAPK). Regulating cell proliferation/invasion, differentiation and survival processes, MAPK pathway represents a potent driver of melanoma development and progression [73]*.* Mutations activating BRAF protein are the most frequent, with the prevalence of the spot mutation causing amino acid substitution of Valine (V) at position 600 in Glutamic Acid (E, V600E) or Lysine (K, V600K) [74]*.* Class I (V600E and V600K), Class II (K601E and G469A) and Class III (G466V and D594N) BRAF mutations differently affect the biochemical behavior of the kinase [75]*.* The other cell pathways most affected in melanoma are neuroblastoma RAS viral oncogene homolog, and NeuroFibromatosis type 1 (NF1). The most modern genomic classification of melanoma derives from The Cancer Genome Atlas Network, which classifies melanoma into 4 subtypes based on the most frequently mutated genes: BRAF (30–35%), NRAS (15–20%), NF1 (20%), triple *wild type* (25%). The absence of mutations in the BRAF, RAS and NF1 genes and mutations and amplifications of c-KIT represent one of the characteristics of this last subtype. Mutations in TERT, TP53, CDKN2A (Cyclin Dependent Kinase), ARID2, PhosphoInositide 3-Kinase (PI3K), Protein Kinase B (AKT), Phosphatase and TENsin homolog (PTEN) and mechanistic Target Of Rapamycin (mTOR) have been also observed [76, 77]*.*

The choice of treatment for melanoma is closely dependent on the stage of the disease (from stage 0/melanoma in situ to stage IV), depending on several factors including the thickness of the tumor and whether melanoma has spread to lymph nodes or other parts of the body. Surgery remains the treatment of choice for the in situ melanoma up to operable stages III [78]*.*

**Fig. 5:**
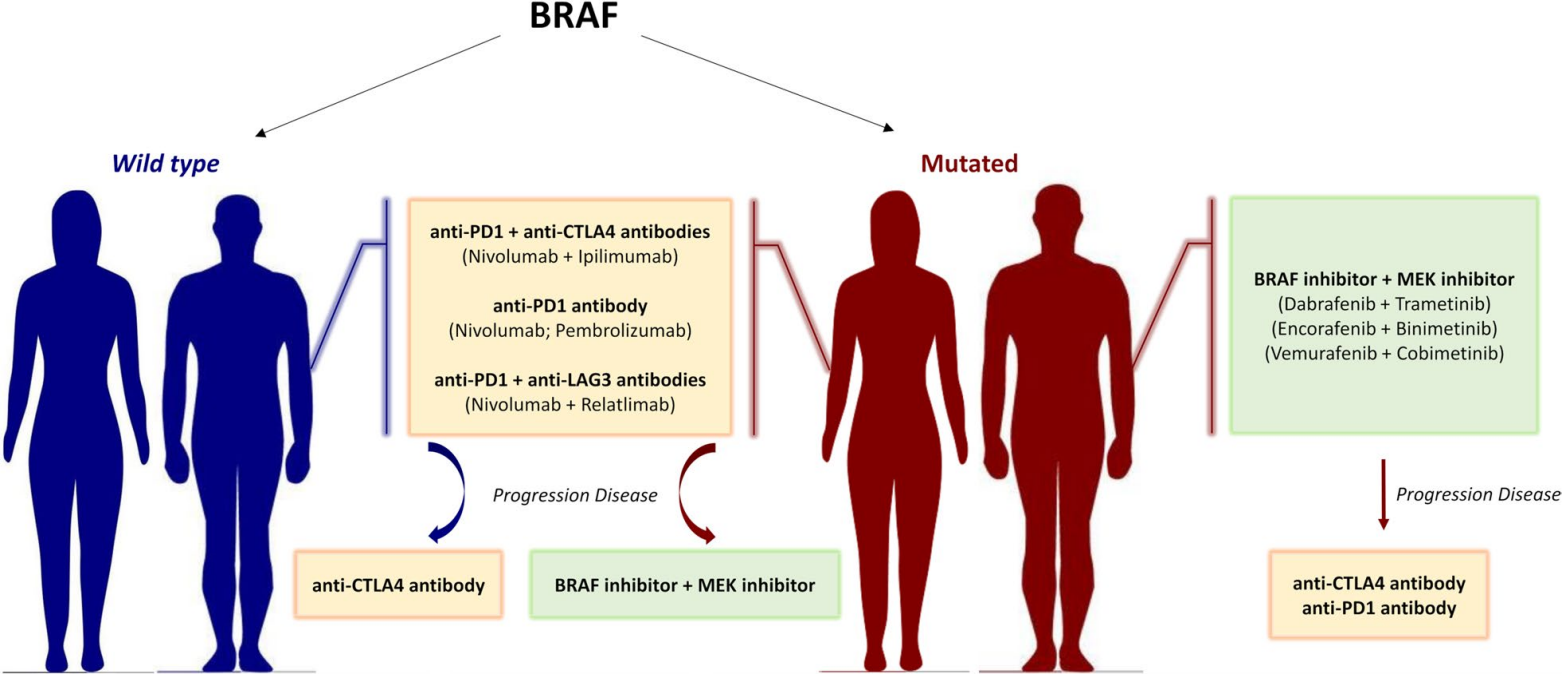
Schematic representation of approved treatments for advanced melanoma

Since 2011, the treatment of advanced melanoma (stage III inoperable and stage IV) has been profoundly revolutionized by the advent of new-generation therapies and, in particular, molecular target therapy and immunotherapy (Fig. 5).

These adjuvant therapies, following surgical resection, stand as common standard first-line treatments and have led to improved clinical outcomes. In fact, although in the past the use of chemotherapy for the treatment of metastatic melanoma was widespread, currently its use is limited, being expected only after the failure of target therapy and immunotherapy [79, 80]*.*

Although more than 300 BRAF missense mutations have been discovered in melanoma patients, the therapeutic choice for the treatment is guided by the presence or absence of mutation in codon 600 of the BRAF gene: V600E, V600K, V600D/G/R/M (aspartic acid, D; glycine, G; arginine, R; methionine, M). These mutations occur respectively in about 85%, 15% and 5% of cases. Food and Drug Administration (FDA) approved drugs only targeting Class 1 BRAF mutants. The V600E mutation represents the strongest predictor of response to the combination of BRAF (Dabrafenib, Vemurafenib, Encorafenib) and MEK (Cobimetinib, Binimetinib, Trametinib) inhibitors, thus identifying patients who may benefit from this treatment [81, 82]*.*

The therapy of melanoma, as well as other solid tumors, has been revolutionized by the advent of immunotherapy. The demonstration that tumors are able to increase the normal pathways that regulate the inhibition of T cells, the so-called immunological checkpoints, has provided new targets for immunotherapy [83]*.* Before 2011, immunotherapy in the treatment of advanced melanoma consisted of the use of high doses of Interleukin 2, with a percentage of objective or complete responses lower than 20%, and high toxicity [84]*.* Starting from 2011, two immunological checkpoints have become therapeutic targets for melanoma: Cytotoxic T-Lymphocyte Antigen 4 (CTLA4) and Programmed Death protein-1 (PD-1). Monoclonal antibodies directed against CTLA4 (Ipilimumab) or PD-1 (Nivolumab and Pembrolizumab) were approved by FDA to activate immune T cells through the blockade of the PD-1/PD-L1 and CTLA4/CD80-86 checkpoint pathways [85]*.* The evidence that activation of the PD-1 and CTLA4 pathways occur at different stages of T-lymphocyte maturation has paved the way for the combinatorial immunotherapy with Ipilimumab and Nivolumab, approved by FDA in 2015.

Another immunological checkpoint that is gaining therapeutic importance is Lymphocyte-Activation Gene 3 (LAG3). Recently, the European Medicines Agency (EMA) approved the use of Relatlimab, an anti-LAG3 monoclonal antibody, in combination with Nivolumab for the first-line treatment of advanced melanoma [86].

In 2024 FDA approved Amtagvi (Lifileucel), the first cellular therapy for the treatment of melanoma patients with unresectable disease, previously treated with other target- or immuno- therapies [87].

New types of treatment are being tested in clinical trials. They include vaccines (NCT01278940), cell therapy (NCT06708455), combination chemotherapy/immunotherapy (NCT04884997), local/systemic combinatorial immunotherapy (NCT03929029) [3], Histone DeACetylase (HDAC) inhibitors alone (NCT02836548) or in combination with immunotherapy (NCT02032810), c-kit inhibitors as single agent (NCT00027586) or with immunotherapy (NCT04546074) [3].

Neoadjuvant treatment, in which surgery follows the immunotherapy, could represent an alternative approach to, or even replace, the adjuvant treatment: several preclinical studies and clinical trials support this possibility [88, 89]*.* In July 2025, Italian Medicines Agency approved the Nadina regimen (Nivolumab/Ipilimumab) for the neoadjuvant treatment of patients with melanoma [90]*.*

Clinical trials to evaluate therapeutic implications of melanoma genomic profiles, tumor biopsy and blood markers are also ongoing (NCT05119829, NCT00348088, NCT04493723) [3].

In recent years, thanks to next generation sequencing and immunohistochemical studies, panels of markers are emerging that consider the mutational load of the tumor, the levels of T lymphocytes, PD-L1, as well as gene signatures able to predict the response to treatment [91, 92].

Despite the progress made in recent decades in the treatment of metastatic melanoma, toxicity, particularly high in elderly patients, representing nearly 50% out of all, as well as resistance to target therapy or immune checkpoint inhibitors (ICIs), represent the main challenges for the effective treatment of the neoplasia. Thus, new potential approaches for overcoming therapeutic resistance are urgently needed as well as new biomarkers predictive of response could allow the identification of patient subgroups for the therapy. In this context, preclinical results obtained with PROTACs could pave-the-way towards their clinical translation for melanoma therapy**.** Some PROTACs demonstrated to overcome resistance to target therapy due to acquisition of NRAS mutation, amplification or kinase domain duplication of BRAFV600 mutation, acquired p61splice variant [93], thus reaching the clinical phase for BRAFV600 mutant solid tumors (NCT05668585) [3].

In this review we extensively searched Pubmed [94] for manuscripts and reviews reporting findings validated in in vitro and/or in vivo preclinical melanoma models. We also described several PROTACs degrading proteins relevant in melanoma pathobiology that have never been studied in melanoma, thus representing future possible PROTACs to be tested in melanoma. A list of PROTACs that advanced to clinical trials [3] has been also reported.

## Protacs in preclinical melanoma models

**Fig. 6:**
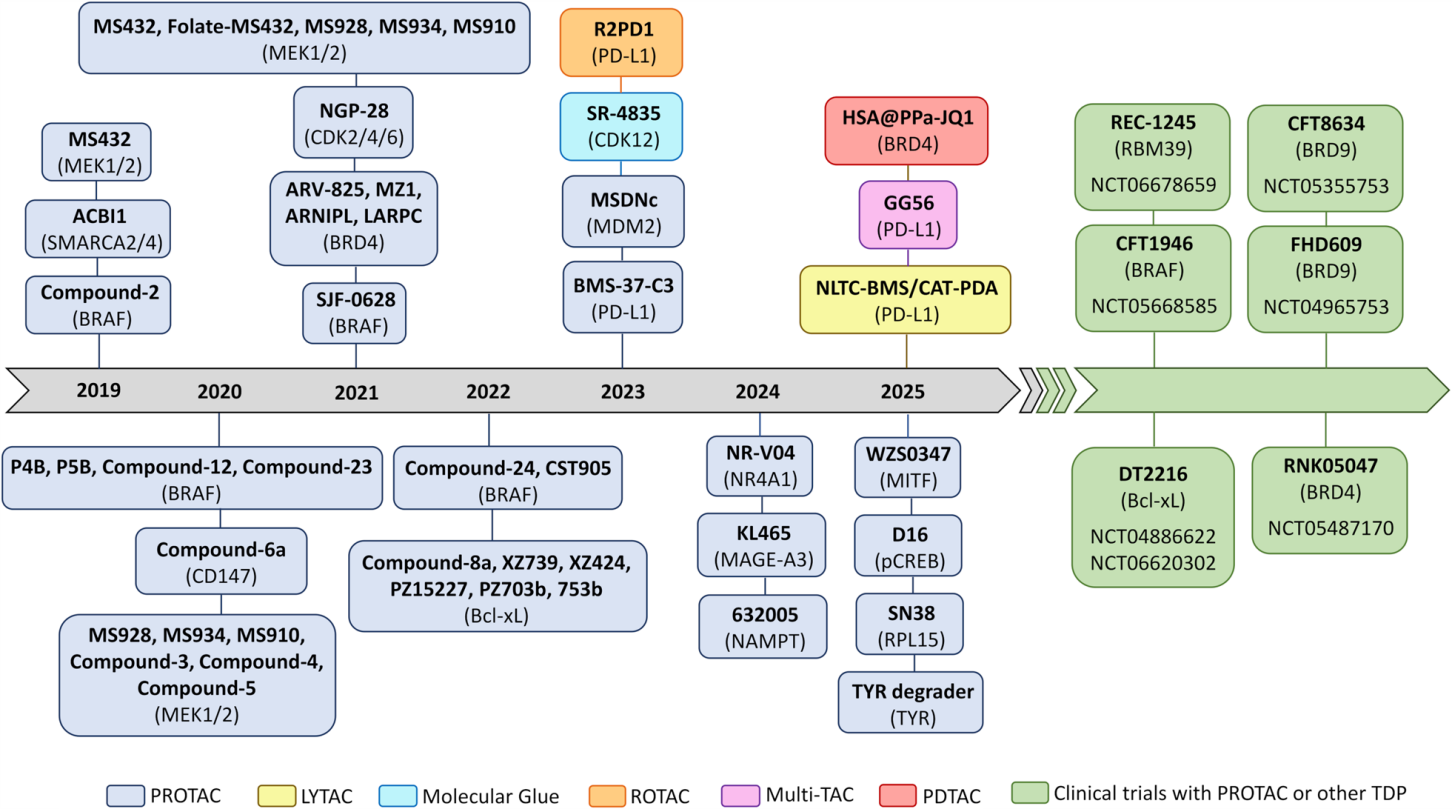
Timeline of preclinical studies with targeted protein degradation (TPD) in melanoma models, and in clinical trials including melanoma patients (NCT05668585, Phase 1/2 in subjects with BRAF V600 mutant solid tumors; NCT04886622, Phase 1 in relapsed/refractory malignancies; NCT06620302, Phase 1/2 in relapsed or refractory solid tumors; NCT05355753, Phase 1 in locally advanced or metastatic SMARCB1-perturbed cancers; NCT04965753, Phase 1 in advanced SMARCB1-loss tumors; NCT05487170, Phase 1/2 in subjects with advanced solid tumors/diffuse large B-cell lymphoma [3])

Several in vitro and in vivo preclinical studies performed in melanoma models reported the antitumor activity or antitumor immunity of PROTACs through the degradation of proteins involved in melanoma pathobiology, response to therapy or immune evasion. PROTACs include degraders of BRAF, BRG1-associated factor (BAF)/SWItch/Sucrose Non-Fermentable (SWI/SNF), BRD4, Nuclear Receptor 4A1 (NR4A1), Melanoma-Associated Antigen 3 (MAGE-A3), Murine Double Minute 2 homolog (MDM2), CD147, Bcl-xL, Nicotinamide Adenine Dinucleotide (Nad)/Nicotinamide Phosphoribosyl Transferase (NAMPT), cAMP response element-binding protein (CREB), MITF, tyrosinase (TYR), Ribosomal Protein L15 (RPL15), MEK1/2, CDKs and PD-L1 proteins (Fig. 6, Table 2).

**Table 2:** PROTACs in melanoma preclinical models and in clinical trials

Target		PROTAC	E3 Ligase	Ligand	Reference	Research status
BRAF		Compound-**2**	CRBN	Rigosertib	97	In vitro
		P4B	CRBN	BI882370	32	In vivo
		P5B	CRBN		99	In vitro
		Compound-**12**	CRBN	Vemurafenib	98	In vitro
		Compound-**23**	CRBN		98	In vitro
		Compound-**24**	CRBN	PLX-8394	101	In vitro
		CST905	VHL		104	In vitro
		SJF-0628	VHL	Vemurafenib	102	In vivo
		CFT1946	CRBN		93	Phase I trial (NCT05668585*)
BAF	SMARCA2/4	ACBI1	VHL	Class VIII bromodomain-ligand	111	In vitro
	BRD9	CFT8634	CRBN	BRD7/9-ligand	110, 111	Phase I/II trial (NCT05355753*)
		FHD609	CRBN		110, 111	Phase I/II trial (NCT04965753*)
BRD4		RNK05047	HSP90	BRD4-ligand	116	Phase I/II trial (NCT05487170*)
		ARV-825	CRBN		120–122	In vivo
		ARNIPL	CRBN	Palmitoyl-DL-carnitine chloride, ARV-825	118	In vitro
		LARPC	CRBN		118	In vitro
		MZ1	VHL	JQ1	128	In vivo
NR4A1		NR-V04	VHL	Celastrol	148	In vivo
MAGE-A3		KL465	VHL	MAGE-A3-ligand	163	In vitro
MDM2		MSDNc	MDM2	MDM2-ligand	170	In vivo
CD147		Compound-**6a**	CRBN	Pseudolaric acid B	191	In vivo
Bcl-xL		XZ739	CRBN	Navitoclax	197	In vitro
		XZ424	CRBN	A1155463	197	In vitro
		PZ15227	CRBN	Navitoclax	197	In vivo
		DT2216	VHL		11, 198, 199	Phase I/II trial (NCT04886622*, NCT06620302*)
		PZ703b	VHL		197	In vitro
		753b	VHL		197	In vitro
		Compound-**8a**	IAP		11	In vitro
NAMPT		632005	CRBN	SIAIS630120	206	In vivo
pCREB		D16	CRBN	Rhein	209	In vivo
MITF		WZS0347	CRBN	TT-012	215	In vitro
TYR		TYR degrader		VH032-Azi + Alk-Tln	220	In vivo
RPL15		SN38	CRBN	SN-38	222	In vivo
MEK1/2		MS432	VHL		223	In vitro
		Folate-MS432	VHL	MS432	225	In vitro
		MS928	VHL		224	In vitro
		MS934	VHL		224	In vitro
		MS910	CRBN		224	In vitro
		Compound-**3**		MEK1/2-ligand	226	In vitro
		Compound-**4**		MEK1/2-ligand	226	In vitro
		Compound-**5**		MEK1/2-ligand	226	In vitro
CDK4/6		NGP-28	CRBN	Ribociclib	232	In vivo
PD-L1		BMS-37-C3	CRBN	BMS-37	243	In vitro

^*^NCT05668585, BRAF V600 mutant solid tumors; NCT05355753, locally advanced or metastatic SMARCB1-perturbed cancers; NCT04965753, advanced SMARCB1-loss tumors; NCT05487170, subjects with advanced solid tumors/diffuse large B-cell lymphoma; NCT04886622, relapsed/refractory malignancies; NCT06620302, relapsed or refractory solid tumors [3]

Among these proteins, antitumor efficacy of PD-L1, BRD4 or CDKs degraders in melanoma also include MG, GlueTAC, LYTAC, PepTAC and ROTAC (Table 3).

**Table 3:** Targeted protein degradation in melanoma preclinical models

Target	Compound	TACnology	References
BRD4	HSA@PPa-JQ1	PDTAC	[126]
CDK12/cyclin K	SR-4835	MG	[55, 56]
PD-L1	BMS-37-C3	PROTAC	[243]
	R2PD1	ROTAC	[63]
	NLTC-BMS/CAT-PDA	LYTAC	[58, 59]
	GG56	MultiTAC	[62]

All compounds are not yet in clinical testing

**Fig. 7:**
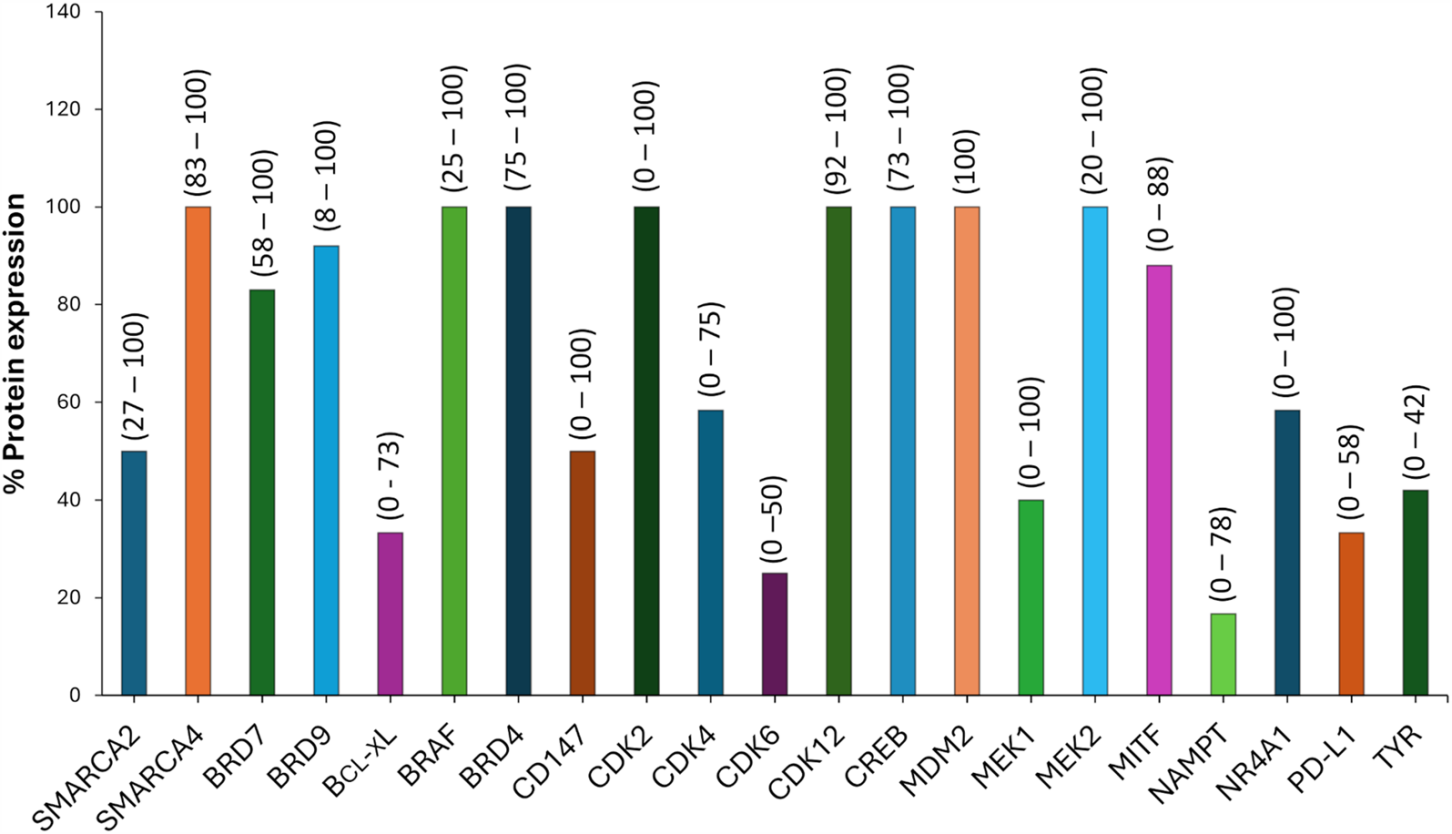
Percentage of protein expression in melanoma specimens from The Human Protein Atlas [289] whose degradation by PROTACs have been demonstrated in preclinical melanoma models, have been considered. In brackets the range of expression (%) of each protein in patients with different solid tumours. Pending cancer tissue analysis for MAGE-A3 and RPL15 proteins

**Fig. 8:**
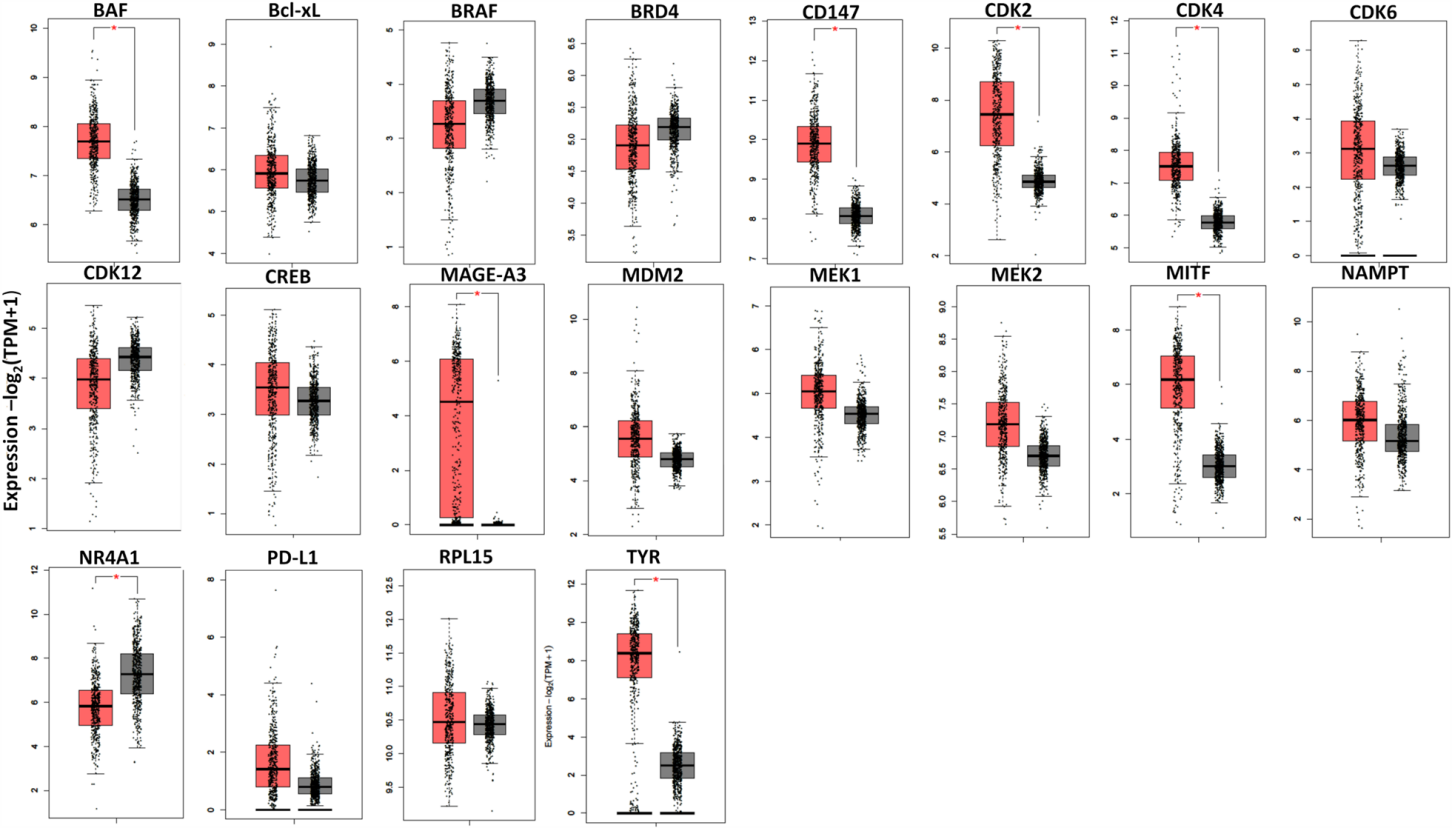
Gene expression in melanoma and normal specimens for proteins whose degradation by PROTACs have been demonstrated in preclinical melanoma models. Graphs have been extracted by TCGA and GTEx data using the Expression Profiling Interactive analysis (GEPIA 2.0 [95]). Box plots show gene expression in melanoma (red, N = 461) versus normal (grey, N = 558) specimens, *p < 0.05

The expression levels in melanoma specimens of all the proteins discussed in this paragraph are reported in Fig. 7. Gene expression in melanoma and normal specimens (extracted by The Cancer Genome Atlas [TCGA] and GTex data, Expression Profiling Interactive analysis, GEPIA 2. 0, http://gepia2.cancer-pku.cn/#index [95]) is shown in Fig. 8. When analysed with the DepMap program (CRISPR screen [96]), while PD-L1 and SMARCA2 genes showed low essentiality (respectively in 0 and 12% melanoma cell analysed), about 90% gene**s** showed high essentiality (from 60 to 100% melanoma cell analyzed). Data about RPL15 gene were not available. In the following paragraphs, each protein will be discussed in terms of functional role in melanoma, inhibitors available and preclinical studies with PROTACs or other TDP, when available.

### BRAF

As reported above, the MAPK pathway is frequently deregulated in melanoma and is involved in melanoma development and progression [73]*.* Together with the advent of immunotherapy, small molecule BRAF kinase inhibitors in combination with MEK inhibitors revolutionized the treatment of metastatic melanoma [79, 80]*.* Often the clinical response is transient and long-term efficacy is restricted by resistance thus, indicating the need of new therapies or new agents able to affect RAS-ERK pathway, targeting not only the BRAF catalytic activity or dimerization, but also BRAF expression, as well as paradoxical activation of MAPK signaling related to RAF dimerization and transactivation.

In this context, the first PROTAC degrading BRAF protein, the compound **2**, was synthesized in 2019 using Rigosertib as warhead. Its ability to degrade the target protein, to reduce the expression of Mcl-1, a downstream protein of BRAF, and to reduce in vitro cell proliferation was reported in one murine melanoma cell line, as well as cancer cells from different origins. IC_50_ values of the PROTAC were similar to those of the mother compound, and cells with highest level of BRAF were the most sensitive to the degrader [97]*.*

One year later, a panel of PROTACs were designed starting from two BRAF inhibitors: Dabrafenib and BI882370. Among them, in vitro and in vivo antitumor activity of P4B was demonstrated in a large panel of human melanoma cell lines carrying V600 BRAF mutations. Mutations included V600D or those responsible of resistance to BRAF inhibitors, such as V600E mutations in which the kinase domain shows a tandem duplication. The authors also suggested altered conformation as a possible mechanism responsible, not only for the reduced interaction with MEK inhibitors, but also for the higher sensitivity to degradation of V600E BRAF mutations when compared to melanoma cells carrying BRAF *wild type* protein. Higher target specificity of P4B when compared to traditional inhibitors was also reported. Of note, P4B displayed enhanced inhibitory efficacy in cell lines showing BRAF mutations conferring resistance to conventional inhibitors, such as BI882370. Moreover, PROTACs based on the BRAFV600E inhibitor, BI882370, were reported to induce higher degradation (DC_50_ 12 nM) than PROTACs based on the potent Dabrafenib inhibitor [32]*,* thus demonstrating the relevance of warhead for PROTACs efficacy.

V600E BRAF PROTACs were also generated from thalidomide, the E3 ligase ligand, using BI882370 and Vemurafenib as BRAF kinase inhibitors: compounds **12** and **23** showed high affinity binding to both *wild type* and V600E BRAF protein but degraded only V600E mutated BRAF protein, thus suggesting the relevance of the protein conformation in allowing the formation of a ternary complex able to degrade the target protein. In accordance with biochemical studies, in melanoma cells harboring V600E BRAF mutation the compounds **12** and **23** induced an impairment of in vitro cell proliferation superimposable to that induced by their respective warheads (Vemurafenib for compound **12** and BI882370 for compound **23**) with IC_50_ ranging from 46.5nM to 500nM [98]*.*

Following these first studies, several PROTACs have been designed to degrade V600E BRAF protein conjugating BRAF inhibitors, such as Encorafenib, Vemurafenib or PLX8394, to different binders through several linkers, and their efficacy have been tested in melanoma models [99–103]*.* Using Encorafenib as ligand for the CRBN binders, pomalidomide, lenalidomide, or VHL ligand-1 through several linkers, Marini and colleagues compared the efficacy of their PROTACs to the effect of Encorafenib or the P5B PROTAC. Even if all PROTACs synthesized showed superimposable inhibition of V600E BRAF kinase activity, with IC_50_ values in the nanomolar range, those with higher stability and cell permeability showed less efficacy in inhibiting cell proliferation of a human melanoma cell line when compared to both Encorafenib and P5B. More importantly, the authors demonstrated PROTACs inefficacy as BRAF V600E degraders, and through computational studies identified linker orientation and mobility as responsible of this failure*,* thus adding new information for the design and synthesis of new PROTACs directed not only against the mutated BRAF protein but also against other proteins [99]*.*

By using BRAF *wild type* and V600E mutated or isogenic melanoma cells with N-RAS or K-RAS mutations, the PLX8394-based CRBN(BRAF)-24 PROTAC has been found to induce V600E BRAF degradation at nanomolar concentrations, and to suppress MAPK signaling with higher efficacy than the BRAF inhibitor PLX-8394. It induced selective inhibition of cell proliferation in melanoma cells harboring V600E BRAF mutations [101]*.* In BRAFV600E/NRAS *wild type* melanoma cell,s the CST905 PROTAC has been reported to degrade BRAF (DC_50_ value 18 nM, and to inhibit melanoma cell viability (IC_50_ 31 nM). CST905 was also found to reduce ERK phosphorylation and to evade the resistance mechanism to first-generation BRAF inhibitors impairing paradoxical MAPK activation, thus indicating longer efficacy and safer profile of PROTACs when compared to first-generation BRAF inhibitors [104]*.*

The antitumor activity of PROTACs has been also reported in mice carrying melanoma models: Alabi et al. evidenced the ability of SJF-0628, a Vemurafenib-based PROTAC, to degrade a panel of BRAF mutants and to trigger antitumor activity in melanoma models with intrinsic or acquired resistance to Vemurafenib. The antitumor efficacy of SJF-0628 was also evidenced in xenograft models harboring the Class II G469A BRAF mutation, without toxic effect to the animals. Both in vitro and in vivo experiments demonstrated a higher efficacy of SJF-0628 in reducing cell viability and tumor growth when compared to Vemurafenib. Notably,* wild type* RAF family proteins were spared by degradation due to the formation of a weak BRAF/PROTAC/VHL ternary complex related to a close/inactive conformation of BRAF protein*.* In agreement with previous findings from Posternak [32], the authors evidenced higher predictive role of protein degradation by the analysis of ternary complex lysates or cells than in vitro results performed with the purified protein [102].

By using cancers from different origin including a panel of melanoma cells, at the beginning of 2026, the potent and selective CFT1946 PROTAC has been reported to overcome limitations of BRAF inhibitors, such as toxicity in normal tissues due to paradoxical activation of MAPK pathway and acquired resistance due to RAF dimer formation. Interestingly, CFT1946 was effective in degrading specifically V600E BRAF mutated protein and inhibiting MAPK pathway in mutated cells sparing normal ones. It also induced in vitro and in vivo antitumor effect in intracranial and in resistant melanoma models with higher efficacy than BRAF inhibitors [93]. Showing drug-like properties, CFT1946 was suitable for clinical development: a phase 1/2 open-label multicenter trial was performed to evaluate its safety and tolerability. The aim of the study was also to determine maximum tolerated dose and the recommended Phase 2 dose of CFT1946 as monotherapy and in combination with Trametinib or Cetuximab in subjects with BRAF V600 mutant solid tumors, including melanoma NCT05668585 [3]. Unfortunately, there are currently no data available regarding the results of this trial, which was completed at the end of 2025.

Very recently, a deep gene expression and pathway-centric enrichment analysis was performed to compare the transcriptomic landscape of BRAF mutated melanoma cells treated with BRAF V600E PROTACs to that of melanoma cells exposed to BRAF inhibitors currently used in clinical practice. Despite the in silico analysis evidenced overlapping transcriptomic changes (about 75%) indicating a superimposable effect of BRAF degradation or inhibition, a specific transcriptomic landscape uniquely corresponding to PROTACs was observed. This includes reduced activity of MAPK and AKT/PI3K/mTOR pathways and early drug resistance, increased cell cycle arrest and expression of apoptotic genes, thus indicating higher efficacy of BRAF degradation compared with BRAF inhibition [103]*.* It should be interesting to compare the two kinds of treatment after longer time exposure to study eventual differences in the adaptive acquisition of resistance to the treatments and to further investigate if PROTACs also show a clinical advantage respect to BRAF inhibitors.

In conclusion, in addition to the currently pursued approaches for the design and synthesis of BRAF ligands (ATP-competitive inhibitors, allosteric inhibitors, multi-target ligands, already known drugs repurposed towards BRAF) [105], all these findings indicate PROTACs as a possible opportunity to expand the possibility of targeting BRAF protein to treat melanoma patients harboring V600E mutations through induction of BRAF degradation and evasion of the paradoxical activation of the RAF-MAPK pathway.

### BAF (SWI/SNF)

BAF (SWI/SNF) complexes have chromatin remodeling functions and, for their involvement in transcriptional regulation, are relevant in melanoma pathobiology and response to immunotherapy [106–108]*.* They are composed by a plethora of different proteins including SMARC-A2-4/C1-2/D1-3, SMARCA4, BRD7-9.

Pharmacological inhibitors of BAF (SWI/SNF) complexes, affecting their catalytic activity or targeting the bromodomains, demonstrated antitumor efficacy in cancers including melanoma [109] and some of them advanced to clinical trials [110].

Several PROTACs have been developed to impact chromatin structure and gene expression through degradation of key subunits of SWI/SNF complex in cancer models including melanoma [110, 111]*.* Some of them progressed in Phase I/II clinical trials for locally advanced or metastatic SMARCB1-perturbed/SMARCB1-deleted cancers (CFT8634/BRD9, NCT05355753; FHD609/BRD9, NCT04965753 [3])*.*

Regarding melanoma, only one paper demonstrated the efficacy of degrading BAF (SWI/SNF) complexes by PROTACs: degradation of the BAF ATPase subunits SMARCA2 and SMARCA4 by the PROTAC ACBI1 evidenced in vitro antiproliferative and proapoptotic functions in SMARCA4-deficient melanoma cells. ACBl1 was designed for targeting the non-functional bromodomain of the two subunits. Effect superimposable to those of Doxorubicin or genetic knockdown was observed. ACBI1 also reduced the expression of PBRM1, a member of the SWI/SNF complex, even if with lower potency, as well the level of other subunits such as ACTB, BCL7A, PHF10 and ACTL6A [111]. This study strongly suggests to further investigate the inhibition of ATPase or other subunits of SWI/SNF complex both in melanoma models and in normal/non-transformed cells.

### BRD4

The epigenetic reader, BRD4, is associated with the genesis, development and resistance to therapy of various cancers, including melanoma, thus representing a possible target for epigenetic therapy. It is induced by BRAF mutation and is upregulated both in primary and metastatic melanoma tissues when compared to nevi and melanocytes [112]. BRD4 belongs to the Bromodomain and Extra-Terminal domain (BET) family (BRD2, 3, 4, 5) and, through its binding to N-ε-acetylated lysine residues in histone and nonhistone proteins, is involved in gene regulation and signal transduction. Several BRD4 specific inhibitors, or less specific pan-BET inhibitors, have been identified and reported to affect tumor growth but, in some cases, lack of specificity restricted their advancement into clinical trials, and none BRD4 inhibitor has been approved for cancer treatment [113–115].

In the last years various BRD4-targeted PROTACs, with higher efficacy and selectivity than BRD4 inhibitors, have been developed. Among them, RNK05047 advanced in a first-in-human phase I/II clinical trial (CHAMP-1) to evaluate safety, tolerability, pharmacokinetics and pharmacodynamics in diffuse large B-cell lymphoma and advanced solid tumors (NCT05487170 [3]) [116]*.* RNK05047 binds to both BRD4 and members of the Heat Shock Protein 90 (HSP90) chaperone protein family. Preclinical data and potential effect of RNK05047 in cancer models are not available [117]*.*

Preclinical studies in melanoma models demonstrated the antitumor efficacy of other PROTACs against BRD4 when administered alone or in combinatorial therapies [118, 119]*.* For a better transport into the cell or into specific organs, the combination of PROTACs with nanoparticles has been designed (nano-PROTACs). These include lipid-, inorganic-, albumin- and polymeric- encapsulated nanoparticles, as well as hydrogel-based or patch-mediated systems [47, 118, 120]*.* To this regard, preformulation studies also allowed the definition of self-nano-emulsifying PROTAC delivery system with better pharmacokinetic of encapsulated (ARNIPL, LARCP) than free (ARV-825) PROTACs against BRD4 both in sensitive and target therapy- resistant melanoma models [118, 120–123]*.* Thus, overcoming some challenging hurdles, as water solubility.

As the tumor suppressor gene, PTEN, is often downregulated in melanoma cells resistant to BRAF inhibition, BRD4 degraders have been also tested in combination with PTEN modulators. ARV-825, free or encapsulated in nanoliposomes, when combined with Vemurafenib or PTEN plasmid or the tyrosine kinase C inhibitor, Nintedanib, induced a synergistic effect in two-dimensional (2D) and three-dimensional (3D) multicellular spheroids of melanoma cells intrinsically resistant to BRAF inhibitors or showing acquired resistance. Cell proliferation/viability, migration, vasculogenic mimicry as well as the expression levels of the “undruggable” c-myc oncogene, directly regulated by BRD4, were strongly affected by the combined treatments*.* Downregulation of proliferation markers and BRD4 protein levels, and induction of necrosis ware also observed in Vemurafenib-resistant melanoma xenograft models after treatment with the ARV-825/Vemurafenib combination. Negligible in vivo toxicity was induced by the treatments [120–122]*.*

The same PROTAC was also co-loaded in PEGylated nanoliposomes, with Palmitoyl-DL-carnitine chloride, an inhibitor of the protein kinase C, a kinase playing pro-tumorigenic role in melanoma [124]. In in vitro studies, these encapsulated liposomes showed antiangiogenic activity in endothelial cells, and anti-proliferative, anti-migratory and anti-vasculogenic mimicry activities in Vemurafenib-resistant melanoma cells [118].

ARV-825 was also employed to construct the magnetic nanoparticles, MNC@Ca/MnCO3/ARV/anti-PD1, in which ARV-825 and anti-PD1 antibodies were loaded in magnetic nanoclusters coated with calcium-doped manganese carbonate. This tumor antigen capture system showed the advantage of inducing in vitro ferroptosis and anti-migratory ability. By using a mouse melanoma model, it was reported to be biocompatible and to induce antitumor activity. Moreover, it showed immunostimulatory effect in terms of maturation/activation of bone marrow-derived cells, enhancement of antigen presentation, induction of immunogenic cell death. Systemic treatment of nanoparticles also reduced the in vivo formation of artificial/experimental metastases [125]*.*

Very recently, a PDTAC nanoplatform has been proposed for light activatable melanoma treatment: both in vitro and in vivo findings evidenced the ability of HSA@PPa-JQ1 to degrade BRD4 specifically at the irradiated tumor site and to induce cell death in melanoma models [126]*.* Even of interest, this spatiotemporal controllable PROTAC needs to be validated in terms of DNA damage induced by light irradiation.

Aberrant activation of components of Hedgehog signaling pathway, such as GLI1 and SMO, occurs in melanoma [127]*.* As BRD4 also acts as cofactor of SOX2 to control the promoter activity and expression of GLI1, studies acting to inhibit the two pathways have been performed. They demonstrated that the BRD4-targeted PROTAC, MZ1, induced preferential degradation of BRD4, over BRD2 and BRD3. It also synergized with the SMO antagonist, MRT-92, both in vitro (2D and 3D) and in vivo (orthotopic) melanoma models, with not significant toxicity in mice. MZ1 also inhibited the self-renewal ability of melanoma stem-like cells, irrespectively to their mutational status. This study highlights the possibility of reducing BRD4 and SMO levels to treat  melanoma patients with high levels of SOX2 and GLI1 proteins [128]*.* By adding a cyclizing linker to MZ1, macrocyclic PROTACs constraining BRD4 in its bioactive conformation, have been synthesized and demonstrated to show cellular activity comparable to MZ1. Unfortunately, their antitumoral efficacy was evidenced in cancer cells not including melanoma [129].

The advantage of using BRD4 PROTACs is also represented by its indirect effect on PD-L1 levels, being PD-L1 a target of BRD4-mediated gene transcription [130]*.* This could allow the amplification of antitumor effect through the effect of BRD4 PROTACs on tumor cells as well as on macrophages, and tumor-associated dendritic cells and, consequently, on anti tumor cytotoxic T cells present in the TME. As reported for inhibitors of BRDs, the specific PROTAC could reduce BRD4 occupancy at the PD-L1 locus with consequent reduction of mRNA production [130].

Although many studies have been performed on the use of BRD4 PROTACs in melanoma models, we believe that much more studies needs to be done in this area. We envisage the analysis of ultrasound-activatable PROTAC prodrugs to degrade the BRD4 protein in melanoma preclinical models, with the final goal of overcoming immunosuppression and inducing immunogenic cell death and facilitating sonodynamic therapy, as reported for pancreatic cancer [38]*.* Future studies are also warranted toward the study of BRD4 DAC in melanoma models. To this regard, by using cancer models not including melanoma, very recently an elegant paper reported on the antitumor efficacy of a DAC in which a BRD4-degrading PROTAC was conjugated with the Receptor tyrosine kinase-like Orphan Receptor 1 (ROR1) antibody [65, 131]. The antitumor efficacy of Intramolecular Bivalent Glues (IBGs), acting as bifunctional degraders of BRD2 and BRD4, should be also investigated in preclinical melanoma models. This approach showed higher affinity and degradation ability, in tumor cells not including melanoma, when compared to conventional bivalent PROTACs and monovalent glues [132].

Considering the previously reported ability of BRD4 to increase the escape of cancer cells from immunosurveillance, to suppress the interferon-gamma (IFN-gamma)- and tumor necrosis factor-alpha-induced expression of PD-L1, and the ability of BET inhibitors to delay melanoma growth in a CD8-mediated manner [133–136]*,* all these studies indicate that targeting BRD4 with PROTACs or small molecule inhibitors, could also potentially represent a tool to induce antitumor immune response in melanoma and other cancer types. These findings could provide a new avenue for melanoma therapy, through the degradation of BRD4 protein or molecules directly or indirectly correlated with its expression/function such as SOX2, GLI1 or PD-L1.

### NR4A1

NR4A family receptors, including NR4A1 (Nur77/TR3/NGFIB), NR4A2 (NURR1), and NR4A3 (NOR-1), act as transcription factors regulating several cellular processes [137]. Among this family, NR4A1 is an orphan nuclear receptor that plays multifaceted roles*.* Extensive preclinical studies have underscored a pro-tumorigenic role of NR4A1 through its “non-genomic” effect [138] on fatty acid oxidation [139]*,* apoptosis [140]*,* autophagy [141], angiogenesis [142], metastatization [143, 144] and immune system [145]*.*

NR4A1 is activated by the BRAF-MEK-ERK cascade in melanoma cells, and its expression contributes to melanoma tumorigenicity and confers resistance to inhibition of the MAP kinase pathway [146, 147]*.* The relevance of NR4A1 in melanoma TME has been also reported: (i) induction of NR4A1 is required for TRAIL-dependent death of melanoma cells and for Natural Killer cells (NK) recruitment by macrophages [144]; (ii) high levels of NR4A1 in melanoma-infiltrating immune cells (regulatory T, exhausted CD8 positive T, B, dendritic and NK cells, as well as monocytes and macrophages) has been found [148]; (iii) NR4A1affects the functions of different immune cell types within the melanoma TME [145]; (iv) the use of melanoma bearing NR4A1 knowckout mice demonstrated NR4A1correlation with immune suppression, thus suggesting NR4A1 targeting to induce immune activation in melanoma therapy [148]*.*

Although several antagonists (Celastrol, CCE9, DIM-C-pPhOH) of NR4A1 have been designed and some chemotherapeutics (Cisplatin) or natural compounds (Kaempferol, Quercetin) have been found to affect NR4A1 functions, the use of NR4A1 inhibitors in the clinic is limited by low bioavailability, adverse effects, moderate binding affinity, and narrow therapeutic window [149]*.*

Very recently NR-V04, a NR4A1-specific PROTAC with an excellent pharmacodynamics and pharmacokinetic behavior, has been designed by using Celastrol as warhead. NR-V04 has been found to inhibit tumor growth in melanoma bearing NR4A1 *wild type* mice, but not in NR4A1^−/−^ mice or mice lacking T, B, and NK cells or strain mice lacking mature B cells or depleted for CD8 positive T cells. Induction of both effector memory CD8 positive T and B cells, as well as reduction of myeloid-derived suppressor cells, have been identified as NR4A1 mechanism of action [148]*.*

Considering that together with other factors belonging to the same family, as NR4A2 and NR4A3, NR4A1 can regulate stemness and persistence of Chimeric Antigen Receptor T cell (CAR-T) limiting their functions both in vitro and in vivo, NR-V04 could also represent a promising approach to generate more active CAR-T cells to use for the treatment solid tumors, including melanoma [150, 151]*.*

In conclusion, NR-V04 could represent a valid therapeutic approach not only for melanoma but also for those tumors in which NR4A1 positively affects different processes such as cell growth and metabolism, apoptosis and metastasis [152–156]. Given the tumor suppressor role that NR4A1 plays in some tumors such as hepatocellular carcinoma [157], and its role as positive prognostic factor within the TME of colon cancer, NR4A1 inhibitors can be suggested only for those cancer types in which NR4A1 plays an oncogenic or negative prognostic role [145]*.*

### MAGE-A3

Due to its oncogenic activity, immunogenicity, and abundant expression in melanoma tissues, MAGE-A3 represents an attractive eligible target for melanoma therapy, including immunotherapy and vaccine-based immune therapy [158]. MAGE-A3 protein expression ranges from 12 to about 70% in pediatric and adult melanomas, respectively*,* with higher expression rate in metastatic tumors when compared to primary ones [159, 160]. Similar trend was also observed at mRNA levels: about 36% in primary melanoma, from 55 to 81% in metastatic melanoma [160]*.*

Although MAGE-A3 is predominantly expressed in mucosal melanoma responding to immune checkpoint inhibitors, the relevance of this biomarker awaits further study to predict the outcome of Immune Checkpoint Inhibitors in melanoma patients [161]. A phase III clinical trial was performed to evaluate the benefit of the MAGE-A3 immunotherapeutic (GSK 2132231A) in preventing disease relapse when given to stage III, MAGE-A3-positive melanoma patients after surgical removal of tumor (NCT00796445). Unfortunately, it has been stopped for adverse events [162]*.*

Very recently, KL465, the first PROTAC degrading MAGE-A3, has been developed and reported to inhibit in vitro proliferation of MAGE-A3 positive cancer cells from different origin, including melanoma, although no more than 50% of cell proliferation inhibition was observed, probably because of MAGE-A3 protein short half-life [163]*.*

As biochemical functions of MAGE-A3 include its ability to act as adaptor of E3 ligase, and consequently regulator of its activity, it could also represent an eligible target for the development of cancer- specific PROTACs. In the future, we hope these studies may serve both to develop new and more potent PROTACs capable of optimally degrading the MAGE-A3 protein, and to create PROTACs directed against a plethora of proteins to be degraded by E3 ligases.

### MDM2

MDM2 represents one of the pivotal factors inhibiting the functions of the tumor suppressor p53, which is expressed in about 5% and 25% of primary and metastatic melanoma, respectively [164]*.* Its overexpression is a frequent feature of cancer cells with *wild type* p53 [165]*.* MDM2 and p53 proteins have been reported to influence each other, and inactivation of MDM2 in *wild type* p53 tumors, or restoration of mutant p53 functions with mutation-specific or pan- inhibitors, have been recognized as a possible approach to eradicate tumor cells, including melanoma [166, 167]. Although several MDM2 inhibitors progressed into clinical trials, their side-effects, mainly represented by hematologic toxicity and low efficacy, hampered their use in the clinic and none of them have been approved by FDA. A first-in-human study demonstrated that Alrizomadlin, a novel MDM2/p53 inhibitor, has an acceptable safety profile and showed promising antitumor efficacy in advanced solid tumors with MDM2-amplification and *wild type *p53 [168]*.*

To overcome the limitations presented by MDM2 inhibitors, in the last years several PROTACs, including peptide-based-PROTACs and Homo-PROTACs utilizing the E3 ligase activity of MDM2, have been designed to achieve MDM2 targeted degradation and p53 reactivation [169]*.* Supraparticles derived from MDM2 self-degradation catalysts, termed MSDNc, have recently been designed to bind and degrade MDM2 protein and to restore p53 functions. Physicochemical and pharmaceutical studies in mice showed no toxicity, as well as proteolytic resistance and biological stability, by these homo-PROTACs. Moreover, when intravenously administered in melanoma-bearing mice, MSDNc determined a significant tumor suppression paralleled by down-regulation of MDM2 expression and activation of the p53 protein. These PROTACs also enhanced the number of cytotoxic T lymphocytes (CD3 positive/CD8 positive activated T cells) while decreased the regulatory ability of T lymphocytes (CD4 positive/CD25 positive T cells) in tumor tissues. More importantly, through p53 restoration, MSDNc showed a synergistic effect, in terms of tumor growth reduction, when administered in allograft model of melanoma in combination with the anti PD-1 monoclonal antibody, thus indicating their potential translation in clinic [170]*.* To extend and validate these studies to other E3 ligases, in-depth analyses are needed from both the biochemical and mechanistic points of view of the ligase of interest.

A very interesting paper conducted in cancer model not including melanoma, surprisingly reported the ability of MDM2-PROTAC (YX-02-030) not only to activate p53 in *wild type* cells but also to stabilize/activate p73 in p53-mutated or -deficient cells. The authors also demonstrated in vitro and in vivo higher activity of these PROTACs than MDM2 inhibitors (Nutlins, RG7112, RG7112D) or other PROTACs degrading MDM2 (MD-222/224, MS3227), whose efficacy has been reported to be strictly related to p53 *wild type* cells [171–173]*.* As p53 mutations, occurring in about 25% of metastatic melanoma, can render melanoma cells insensitive to MDM2-PROTACs, these data open the possibility of using YX-02-030 to treat p53-inactivated melanoma. Further studies are needed to analyze the addiction of p53-deficient melanoma cells to MDM2.

Finally, considering the recently reported ability of MDM2 inhibitors (HDM201, RG7112) to abrogate the immunosuppressive melanoma TME in a p53-dependent manner [174]*,* the immunosuppressive activity of different MDM2-PROTACs in preclinical models of melanoma should be also investigated.

### CD147

CD147, also known as basigin, extracellular matrix metalloproteinase inducer and HAb18G, is a glycosylated transmembrane protein belonging to the immunoglobulin superfamily and functioning as a multifunctional molecule binding several partners [175, 176]*.* It is overexpressed in a wide range of cancers from different origin, including melanoma, where it is associated with poor prognosis, represents an independent prognostic biomarker and plays a pivotal role in tumor progression, metastatization and resistance to target therapy [177–181]*.* Moreover, CD147 translocation to the mitochondria in advanced melanoma is associated with the invasion of melanoma cells [182]. CD147 also represents a tumor target in the context of NK as well as CAR-T and CAR-Macrophages therapy [183]*.* These findings suggest CD147 as a powerful in the therapy of melanoma in the context of high levels of CD147 expression [184–187].

Even if anti-CD147 monoclonal antibodies have been used for the treatment of patients with hepatocellular carcinoma [188], only preclinical data are reported on the possibility of inhibiting CD147 expression/function in melanoma. In particular, the efficacy of anti-CD147 monoclonal antibody (MEM-M6/1) in melanoma models has been evidenced in terms of induction of cell death via the impairment of glycolytic energy metabolism [189]*.* Moreover, specific inhibition of interaction between CD147 and other receptors has been identified as a novel therapeutic strategy for a subset of melanoma [190]*.*

Starting from the Pseudolaric acid B, a natural antagonist of CD147, the first and only PROTAC degrading CD147, compound **6a**, has been developed and exclusively tested on preclinical melanoma models. It degraded CD147, inhibited cell migration and invasion, affected CD147 downstream pathways (metalloproteinase 9 and signal transducer and activator of transcription 3) and inhibited in vivo tumor growth [191]*.* Since one limitation of this work is that all experiments were conducted exclusively on two melanoma models, it would be desirable to extend these studies to a wider panel of melanoma cells, as well as to hematological and solid tumors, for which the expression of CD147 has been found as a clinicopathological and prognostic indicator with therapeutic potential [192–194]*.*

### Bcl-xL

Beyond their involvement in apoptosis regulation, Bcl-2 family proteins play a pivotal role in other cellular processes, including drug resistance and senescence, frequently induced by antineoplastic agents. These two pathways are interconnected as senescent cells are often resistant to apoptosis but paradoxically hypersensitive to BH3 mimetics, small molecules inhibiting pan or specific antiapoptotic proteins [195, 196]*.*

Preclinical studies highlighted the in vitro and/or in vivo antitumor effect of PROTACs degrading one or more antiapoptotic proteins belonging to the Bcl-2 family [197]*.* To reduce platelets toxicity induced by the BH3 mimetic Navitoclax, several PROTACs have been developed using CRBN (XZ739, XZ424, PZ15227) or VHL (DT2216, PZ703b), whose expression is very low in platelets, as well as Inhibitor of Apoptosis (IAP) (compound **8a**). ABT-263 was used as warhead for the design of DT2216, PZ703b, 753b, XZ739, PZ15227, and compound **8a**), while A1155463 was employed for the synthesis of XZ424.

In 2022 a study highlighted the ability of compound **8a** to specifically degrade the Bcl-xL protein, without affecting the expression levels of the Bcl-2 and Mcl-1 proteins, in a human melanoma cell line [11]. More recently, Alcon and colleagues and our group reported on the role of the PROTAC DT2216 against Bcl-xL protein in melanoma models. Despite the higher binding affinity for Bcl-2 than for Bcl-xL protein, the PROTAC DT2216 formed a ternary complex only with Bcl-xL, thus specifically degrading this protein [11]. Alcon’s group evidenced the dependence of senescent melanoma cells to Bcl-xL protein through its interaction with the proapoptotic proteins HRK and Bax, making Bcl-xL an outstanding target for senescent cells and/or considering the possibility of using agents able to avoid HRK downregulation to remove melanoma senescent cells. Specific BH3 mimetics (A-1331852) or PROTAC (DT2216) have been reported to induce senolytic activity against therapy-induced senescence [198]*.* By using a panel of BRAF *wild type* and mutated human melanoma cells, our findings demonstrated the ability of DT2216 to increase the efficacy of target therapy in in vitro and in vivo melanoma models regardless BRAF status [199].

Together with (i) our data demonstrating the in vitro and in vivo induction of tumor progression associated properties by Bcl-xL and Bcl-2 proteins [200–202], and (ii) findings evidencing knock down of Bcl-xL in tumor-infiltrating Treg and dendritic cells after DT2216 treatment, as well as activation of tumor-infiltrating-CD8 positive T cells in tumors not including melanoma [203], our findings and those of Alcon’s group strongly support further investigations on the possibility to translate in clinic the use of antiapoptotic protein inhibitors for solid tumors for their ability not only to kill cancer cells, but also to regulate the immune system. While a phase I, with no available results, evaluated the safety, tolerability, pharmacokinetics and clinical activity of DT2216, in patients with relapsed or refractory malignancies (NCT04886622 [3]), two clinical trials are recruiting patients with (i) relapsed or refractory solid tumors for testing the addition of DT2216 to the topoisomerase I inhibitor, Irinotecan (phase I/II, NCT06620302 [3]) or (ii) recurrent Platinum-resistant ovarian cancer to evaluate DT2216 in combination with Paclitaxel, which is approved by FDA as a treatment option for ovarian cancer (phase Ib, NCT06964009 [3]). We hope these trials can provide useful information and a new avenue for the treatment of melanoma, or more generally, of tumors whose survival is strictly linked to Bcl-xL expression.

### NAMPT

The enzyme co-factor NAD is essential in multiple cellular processes [204]*.* Its biosynthesis can be catalyzed by NAMPT, which represents a potential target for cancer treatment. Overexpression of NAMPT pathway is reported in several cancers including melanoma, where it is considered a driver of drug resistance [205]*.* Despite in recent years, several specific inhibitors of NAMPT have been designed, and some of them are currently in phase II/III clinical trials for melanoma treatment administered alone or in combination (NCT00432107, NCT0072484 [3]), none of them are currently employed in clinical settings.

Modifying PROTACs able to degrade both intracellular and extracellular NAMPT, a next-generation NAMPT degrader, 632005, with high bioavailability, selectivity, half-life and clearance has been identified. It has been demonstrated to induce in vitro antiproliferative and in vivo antitumor effect in NAMPT positive/Nicotinate Phosphoribosyl Transferase (NAPRT)-deficient cancer models including melanoma, thus indicating that patients with these characteristics could beneficiate from the treatment [206]*.* Despite Nicotinic Acid (NA), which is converted in NAD^+^ by NAPRT, has been reported to compromise the efficacy of NAMPT inhibitors in NAPRT-deficient tumor models [207] and supplementation of NA with NAMPT inhibitors resulted in loss of the in vivo efficacy in NAPRT1-deficient tumor models [207]*,* in the study of Zhu and colleagues, NA co-administrated with 632005 strongly reduced in vivo tumor growth of melanoma and other cancer types thus, indicating, the need of further investigations to analyze these controversial results. Surprisingly, no reference has been made by the study of Zhu and coworkers regarding the expression levels of NAMPT/NAPRT in the melanoma model in which the combination 632005/NA was reported to induce in vivo antitumor effect. Thus, we envisage further preclinical studies should be also focused on evaluating the relevance of NAMPT degradation in NAPRT-deficient melanoma models.

### CREB, MITF and TYR

CREB pathway is a crucial signaling cascade in melanogenesis. Following UV exposure or stimulation with α- Melanocyte Stimulating Hormone, its phosphorylated form (pCREB) acts as a key activator of expression of MITF, which in turn increases the expression of target gens, like tyrosinase (TYR) [208]. CREB is a key transcription factor that promotes melanoma growth and metastasis, by activating pro-survival and pro-invasion genes while repressing tumour suppressors such as CYR61 (CCN1) [209]. Recently, D16 PROTAC has been synthesized, based on the structure of Rhein from traditional Chinese herbs, showing reduced cytotoxicity in mouse melanoma cells. However, the authors reported the D16 ability to bind and degrade pCREB in vitro and to suppress melanogenesis in mice [209]**.**

MITF plays a relevant role within the oncogenic landscape of melanoma: its expression is higher in metastatic melanoma when compared to primary tumors, it contributes to drug resistance, controls tumor progression through its effect on NK functions and contributes to the response of autologous T cells to melanoma [210–214]. Recently, WZS0347, a first-in-class PROTAC degrading MITF has been tested in vitro in several melanoma cell lines. It reduced MITF protein levels, downregulated MITF downstream target genes, and inhibited cancer cell colony formation and migration capacity [215]. Considering the relevance of this transcription factor in melanoma development and growth, and the antitumor activity of MITF inhibitors alone or in combinatorial therapies [216, 217], PROTACs degrading MITF [218] should be further investigated in melanoma models, also in consideration of MITF emerging role as a pivotal regulator of the immune response [219].

TYR shows up-regulated activity and expression in malignant melanocytes, offering an effective target for developing diagnosis methods and treatment strategies against skin diseases. In a recent study, the authors designed a multifaceted strategy to leverage the catalytic activity of TYR to generate therapeutics in situ through the “click chemistry", a process based on Copper(I)-catalyzed Azide-Alkyne Cycloaddition (CuAAC) reaction [220]. The resulting TYR-catalyzed in situ-formed PROTACs, effectively degraded TYR in the melanocytes, decreased the melanin levels, and alleviated skin hyperpigmentation in a mouse model. More importantly, anti-cancer prodrugs (Pd1, Pd2) were activated in situ by TYR catalysis to overcome the drug resistance in 3D spheroids and mouse melanoma models [220].

### RPL15

The expression of the ribosomal protein RPL15 60S has been reported to be associated with secretion of Damage-Associated Molecular Patterns (DAMP) and antitumor immune activation in melanoma models. Knockdown or inhibition of this protein sensitized a murine melanoma model against PD-1 blockade through DAMP secretion, enhancement of cytotoxic T lymphocytes and decrease of regulatory T cells [221]. The same group also evidenced that, in addition to its ability to degrade RPL15 nuclear protein, SN38-PROTAC increased the efficacy of anti-PD1 antibody in mouse melanoma models through activation of cGAS-STING signaling in dendritic cells. On the contrary, SN38-PROTAC as single administration was not able to affect melanoma growth [222]. These results indicate RPL15 relevance for melanoma therapy in combinatorial strategies.

### MEK1/2

As above described, combinatorial therapies including BRAF and MEK inhibitors have been approved by the FDA for the treatment of BRAF-mutated melanoma patients [79, 80].

The first-in-class PROTACs inducing the degradation of MEK, MS432, have been designed based the PD0325901 MEK inhibitor. Although in general the inhibition of the phosphorylation of ERK1/2 with the PROTACs was less effective in comparison to a small-molecule inhibitor, the best PROTACs were more effective in inhibiting proliferation of melanoma cells than the inhibitor [223]. By using both VHL and CRBN E3 ligases and different linkers, the same group also designed and tested the efficacy of a variety of PROTACs (MS928, MS934, MS910) in terms of MEK1/2 degradation, inhibition of downstream signaling and suppression of cell proliferation. Among these, even if MS934 showed higher in vitro efficacy and better pharmacokinetics in mice than the previously designed PROTACs, it only moderately potentiated the antitumor efficacy of BRAF (PLX4032) or PI3K (ZSTK474) inhibitors [224]. To achieve targeted degradation of MEK in malignant cells versus noncancerous normal cells, the same group employed a cancer cell selective delivery strategy for PROTACs by conjugating a folate group to a ligand of the VHL E3 ligase. The author demonstrated that among the folate-PROTACs, the folate-MS432 was capable of degrading MEKs in a folate receptor-dependent manner in melanoma cells without increasing the cytotoxicity compared to MS432 [225]. Vollmer’s group also designed and synthesised MEK PROTACs (degrader **3**, degrader **4**, degrader **5**). The group demonstrated the effect of these PROTACs on downstream regulatory pathways (i.e. cytokines release, ERK1/2 phosphorylation) and proliferation of melanoma cells [226]. The efficacy of a SM-CMAD (KFERQ + Cobi-θθ) in degrading MEK1 and MEK2 was evidenced in a melanoma cell line in a concentration- and time- dependent manner, without further investigation of downstream functional assays [72].

One limitation of the results reported on MEK PROTACs is that the three research groups focused their study exclusively on a single melanoma cell line without any validation in a broader panel of melanoma cell lines.Moreover, in vivo  antitumor activity studies were not performed.

### Degradation of CDKs in melanoma by different TPD

Overexpressed or abnormal activated in several tumor types, CDKs are a class of more than 15 multifunctional proteins involved in cell division and proliferation, as well as in DNA repair, transcription, differentiation, apoptosis and drug resistance [10, 227]*.* Due to their role in these processes, several pan inhibitors have been developed and some of them reached the clinical phase: this is the case of CDK4/6 inhibitors (Palbociclib, Ribociclib, and Abemaciclib) which have been FDA-approved alone or in combination therapy for breast cancer patients [227, 228]*.*

The expression of CDK is a frequent event in aggressive melanoma and is associated with the sensitivity to CDK inhibitors [229]*.* Several clinical trials are ongoing to evaluate the safety/tolerability/pharmacokinetic profile of CDK inhibitors or their efficacy in combination with BRAF and MEK inhibitors in patients with BRAF mutated metastatic melanoma (NCT02671513, NCT04720768) with no available results. Due to primary or acquired resistance the efficacy of CDK inhibitors is often limited over time.

Using FDA-approved CDK4/6 inhibitors and different E3 ligases (CRBN, VHL, IAP, MDM2) several PROTACs inducing selective degradation of CDK6 sparing other members of CDK family (BSJ-03-123, compound **6,** CP-10, CST651) or inducing pan degradation of both CDK4 and CDK6 (BSJ-05-017, BSJ-03-096) have been developed. Some of them were more potent than catalytic inhibitors in preclinical models of cancers from different origin, not including melanoma [24, 227, 230, 231]*.*

Two studies reported on the possibility of degrading CDKs in melanoma models through PROTACs or MG. Starting from Ribociclib derivatives, an oral bioavailable PROTAC degrading CDK 2/4/6 proteins (NGP-28) demonstrated in vitro and in vivo antitumor activity in human and mouse melanoma models, through activation of caspase-dependent apoptosis. NGP-28 recapitulated the activity of CDK inhibition, including reduction of  retinoblastoma protein expression/phosphorylation, cell cycle arrest in G1 phase, and anti-proliferative effect [232]*.*

Degradation of cyclins can be also induced by drugs: in 2023, the group of Roux demonstrated that the pharmacological inhibitor of CDK12, SR-4835, in addition to its ability to inhibit RNA Polymerase II phosphorylation, also acted as a MG able to promote CDK12 interaction with Damaged DNA-Binding protein-1 (DDB1). SR-4835 was also reported to degrade cyclin K thanks to the recruitment of the CDK12/cyclin K complex to the CUL4/RBX1/DDB1 ubiquitin ligase. The authors evidenced that cyclin K degradation substantially contributes to the cytotoxicity induced by SR-4835 in BRAF mutated melanoma models, showing high CDK12 activity [55, 56].

As reported for CDK inhibitors, these studies pave the way towards new therapy for the treatment of BRAF mutated melanoma patients and strongly suggest the possibility of evaluating the safety and efficacy of CDK degraders in clinical trials.

### Degradation of PD-L1 in melanoma by different TPD

Several approaches, including PROTACs, ROTACs, LYTACs and GlueTACs, have been reported to degrade PD-L1, an immune checkpoint expressed in the cell surface of tumor and antigen presenting cells. PD-L1 levels increase in these cells during immunotherapy, thus representing an adaptive mechanism of resistance: through the interaction with its partner PD-1, PD-L1 mediates tumor immune evasion, thus representing a prominent target for cancer therapy [233, 234]*.*

As we previously underlined, one limitation of PROTACs is represented by the difficulty to degrade non-cytosolic proteins and, as consequence, most of the proteins degraded by these compounds belong to the intracellular category. This is related to the fact that PROTACs depend on the proteasomal pathway for their mechanism of action [6]*.* Despite this limitation, PROTACs degrading membrane proteins such as PD-L1 have been designed [235–238].

Even if the effect of several PROTACs degrading PD-L1 protein has been evaluated in cancer cells from different origin [239–241], to the best of our knowledge only one paper reported on the activity of a PD-L1 PROTAC on melanoma models. Specifically, CRBN-ligand-based, BMS-37-C3, was synthesized by using the PD-L1 inhibitor, BMS-37 [242] as PD-L1 ligand, and selected among four different E3 ligase (VHL, CRBN, cellular Inhibitor of Apoptosis Protein [cIAP] and MDM2). It demonstrated to reduce PD-L1 expression levels on cell membrane in human and mouse melanoma models and to enhance the killing activity of T cells when co-cultured in vitro with tumor cells, with an effect higher than that induced by BMS-37 [243]*.*

As previously mentioned, the limitation of PROTACs in degrading cell membrane proteins can be overcome by other TPD technologies, involving lysosomes for their degradation. Conjugation of PROTAC with antibodies recognizing both membrane proteins and E3 ligases (RNF43, ZNFR3) with extracellular domains could allow the increase of protein specificity, on-target degradation activity and could overcome undesirable pharmacokinetic properties [64, 244, 245]*.* In 2023 the group of Sun and coworkers developed the ROTAC R2PD1 to direct PD-L1 toward lysosomal degradation. They demonstrated R2PD1 ability to degrade its target (50–90% degradation) and to inhibit tumor growth in melanoma models, through reactivation of cytotoxic T cells, with an effect higher than that induced by a monoclonal antibody targeting PD-L1. The authors also speculated that expression levels of ZNRF3/RNF43 E3 ligases could be responsible of the different kinetics of PD-L1 degradation observed in the different models used [63]*.* As the findings were limited to three established melanoma cell lines with BRAF mutation (V600G), we envisage this study will be extended to *wild type* melanoma models, to whom target therapy cannot be administered but may benefit exclusively from immunotherapy. Primary melanoma cells, as well as cell lines derived from cancers of different origins, for which immunotherapy is currently used in clinical practice, should also be tested with these TDPs [246]*.*

By using the small molecule inhibitor targeting PD-L1, BMS-8, and the cation-independent M6PR to engage endocytosis, at the beginning of 2025 Liu and coworkers developed NLTC-BMS/CAT-PDA, a LYTAC PD-L1 nanocapsule [58]. To reduce immunosuppressive hypoxia and inflammation present within TME, NLTC-BMS/CAT-PDA included catalase. Moreover, to facilitate tumor-selective PD-L1 degradation and to increase tumor accumulation, it was coated with a pH-responsive PEG. The authors demonstrated its efficacy in in vitro and in vivo melanoma models: intravenous injection of NLTC-BMS/CAT-PDA in syngeneic mice was able to inhibit tumor growth (about 90%), recurrence, and lung metastasis in a rechallenge tumor melanoma model. Degradation of PD-L1 protein, decomposition of H_2_O_2_ into O_2_ in tumor tissues, as well as increased T cell-based antitumor immunity was also observed and demonstrated to be responsible of NLTC-BMS/CAT-PDA antitumor activity.

A recently published paper reported on the possibility to degrade PD-L1 by LYTACs in a space- and time- controlled manner to reduce damage to normal tissues. Specifically, photothermal-sensitive bacteria expressing transferrin receptors/LYTAC complex were engineered to be activated in mice bearing subcutaneous melanoma by a smartphone-controlled laser switch and temperature. Tumor growth inhibition and reduction of PD-L1 expression in tumor tissues were observed after treatment with transferrin receptors/LYTAC complex and laser exposure, without dermal damage or systemic toxicity, thus suggesting this platform for future telemedicine [59].

PD-L1 degradation in murine melanoma cells has been also demonstrated by transferrin receptor mediated internalization (TfR-LYTACs). Surprisingly, when intravenously injected in melanoma carrying mice, TfR-LYTACs did not affect tumor growth, due to their poor tumor penetration and short half-life. This limitation was overcome by engineered bacterial Outer Membrane Vesicles (OMV-LYTACs), in which TfR-LYTACs were fused to OMVs: combination of PD-L1 degradation with the immune stimulation by OMVs was able to reduce tumor growth through induction of necrosis and apoptosis. Immune activation (increase of blood NK cells, tumor macrophages and serum/tumor pro-inflammatory cytokines) was also evidenced [247]*.*

More recently, Xiao Y et al. [67] highlighted that v9x, a transferrin receptor and PepTAC targeting PD-L1 was able to degrade PD-L1 both in anti-PD-1-resistant mouse melanoma model and in immune cells. V9x also induced an in vivo antitumor immune response (increase of amount and functions of tumor-infiltrating CD8 positive T cells, enhancement of IFN-gamma by T cells, decreased expression of PD-L1 on tumor-infiltrating dendritic cells and macrophages as well as tumor cells) higher than that observed after treatment with anti-PD-L1 antibody, without sign of toxic effects. By using an orthotopic glioma model, the authors also demonstrated the ability of PepTACs to cross the blood–brain barrier and to increase the survival of mice. Considering that brain metastases occur in almost 50% of melanoma patients, we envisage this approach to be also tested in in vivo preclinical models with melanoma brain metastasis.

Nanobody-based GlueTACs have also been reported to induce internalization and degradation of PD-L1 and in vivo antitumor activity when intratumorally injected in immunocompromised mice carrying a human melanoma model stably expressing PD-L1/EGFP proteins subcutaneously injected with peripheral blood mononuclear cells (ratio 1:1). Of note, PD-L1 degradation by GlueTAC showed higher activity than blocking functions of PD-L1 with the inhibitor, Atezolizumab [61]*.*

In vitro and in vivo antitumor activity of tumor selective PD-L1 degradation in mouse melanoma models has very recently been reported by GG56, a MultiTAC incapsulated into micellar nanoparticles to increase cellular uptake. Employing acid-responsive elements integrated with hydrophilic PEG segment and a PD-L1 inhibitor (BMS-1), GG56 was designed to trigger protonation and dePEGylation, respectively, by extracellular tumor acidity and metalloproteinase-2, with consequent degradation of PD-L1 through endocytosis/lysosomal pathway. GG56 was reported to be more potent than the antibody targeting PD-L1 in reducing tumor growth and PD-L1 expression in tumor membranes and in recruiting tumor infiltrating cytotoxic T lymphocytes [62]*.*

Despite most of these approaches/degraders show the limitation of potential uncontrolled degradation and systemic toxicity, when compared with therapeutic antibodies, which antagonize the interaction between the extracellular receptors and their ligands with consequent block of oncogenic signaling, they represent new strategies for the elimination of surface receptor signaling by degradation. Noteworthy the fact that PD-L1 also shows ligand-independent activity, supports the relevance of eliminating, rather than inhibiting, this receptor to completely block its functions.

All these data provide valuable insights into how PROTACs, and more il genera TPD technology, might be used to increase tumor immune responses through the degradation of specific proteins involved in immune checkpoint regulation.

## PROTACs degrading proteins relevant in melanoma pathobiology that have never been studied in melanoma

Considering the resistance to currently available therapies frequently observed in melanoma patients, novel therapeutic approaches could allow improvement of melanoma patient survival. In the future, it would be desirable to conduct studies with currently available PROTACs whose efficacy on melanoma has not yet been demonstrated, but whose target proteins have been found to play a key role in the pathobiology of melanoma or response to therapies (Table 4).

**Table 4:** Proteins important in melanoma pathobiology and relative PROTACs never tested in preclinical melanoma models

Target	PROTAC	E3 ligase	Ligand	References
SIRT2	Compound-**128**	CRBN	Thiomyristoyl	[251–254]
	Compound-**129**	CRBN		
	TM-P4-Thal	CRBN		[251–254]
	PRO-SIRT2	CRBN		[251–254]
HDAC	FF2039	CRBN	DCAF11-ligand	[251–254]
	JPS016	VHL	Benzamide	[251–254]
BRD2	SIM1	VHL	MZ1, MT1	[255]
WDR5	MS33	VHL	Phenylbenzamide	[260]
	MS67	VHL		[260]
	MS169	VHL		[260]
	NS40	CRBN		[260]
	7Q2	VHL		[260]
TC-PTP	TP1L degrading TC-PTP	VHL	TC-PTP-inhibitor	[262, 263]
	DU-14	VHL	TC-PTP/PTP1B-ligand	[262, 263]
MIF	MD13	CRBN	MIF-ligand	[269]
IDO1	NU223612	CRBN	BMS-986205	[271, 272]
	NU227326	CRBN	NU223612	[271, 272]
	Compound-**2c**	CRBN		[271, 272]

These “speculative” PROTACs include Sirtuin 2 (SIRT2), HDAC, BRD2, WD Repeat Domain 5 (WDR5), TC-PTP, MIF, Indoleamine 2,3-Dioxygenase 1 (IDO1). Considering the relevance of TME in melanoma progression and response to therapy, further research in PROTACs affecting the expression of proteins relevant for the function of cells within the TME are also expected.

Despite several studies demonstrated that Sirtuin 2 (SIRT2) inhibitors, and more in general HDAC inhibitors, could represent a promising strategy for cancer patients, none of these studies analysed the efficacy of PROTACs degrading these proteins in melanoma models [248, 249]*.* SIRT2 belongs to a family of NAD^+^-dependent HDAC [250]*.* In addition to SIRT2, other sirtuins or HDAC are involved in melanoma survival thus, we strongly believe that the study in melanoma models of PROTACs specifically degrading SIRT2 (compounds **128** and **129,** TM-P4-Thal, PRO-SIRT2) or pan-HDAC (FF2039, JPS016) could lead to the optimization of melanoma patient outcomes [251–254]*.* In support of our hypothesis, several clinical trials with HDAC inhibitors alone (NCT02836548 [3]) or in combination with immunotherapy (NCT02032810 [3]) are ongoing in melanoma patients.

The antitumor efficacy of a trivalent PROTACs, SIM1, engaging two domains of BRD2 has been reported to overcome the issue of unfavorable ternary complex frequently encountered, and to improve degradation efficiency in multiple cancer cells not including melanoma ones. It showed high cellular stability and residence time. “Positive cooperativity” among the components of the ternary complex has been demonstrated as a relevant factor for improved degradation efficiency [255]. We, thus, also envisage the study of SIM1 in melanoma models. In fact, in addition to BRD4 [112], also BRD2, belonging to the same family, is overexpressed in primary and metastatic melanoma samples when compared to nevi, and plays a role in melanoma cell proliferation [255, 256]*.*

As part of different complexes, the nuclear scaffolding protein WDR5, is involved in several pathways, including methylation and acetylation of histones [257], thus contributing to oncogenic processes, and to transcriptional regulation of several factors (i.e. PD-L1 and PD1). WDR5 also plays a role in the response of melanoma patients to checkpoint immunotherapy, showing non-responder patients high levels of this protein [258]*.* Thus, inhibiting WDR5-based epigenetic functions or targeting the interaction of WDR5 with other factors, including the previous mentioned BRD4 [259] could open new avenue for melanoma therapy. An elegant paper recently carried out in leukemia cells reported on the possibility of using a ternary complex composed by a PROTAC degrading exogenous and endogenous WDR5 [27]. We envisage the in vitro and in vivostudies of this or other WDR5 PROTACs (MS33, MS67, MS169, NS40 and 7Q2), in solid tumors, including melanoma, or in cancers expressing high levels of WDR5 such as breast, bladder and lung cancers [260]*.*

Another possible strategy to investigate in melanoma models is furnished by degraders of T-Cell Protein Tyrosine Phosphatase (TC-PTP), whose depletion has been reported to enhance the response to immunotherapy in melanoma models through induction of tumor cell antigen presentation, and increase of T-cell receptor signaling, proliferation, as well as activation of T cells [261]*.* To this regard, TP1L degrading TC-PTP and DU-14, dual degrader of both TC-PTP and Protein Tyrosine Phosphatase 1B (PTP1B), two negative regulators of T-cell activation, showed in vitro and in vivo antitumor activity in a panel of cancer models, not including melanoma, as well as increased killing efficiency of CAR-T cells. The ability of DU-14 to induce PTP1B/TC-PTP degradation and to increase pSTAT1/Y701 in a mouse melanoma cell line has been reported, with no further studies regarding its in vitro and in vivo antitumor activity [262, 263]*.* Supported by the findings that TC-PTP/PTP1B inhibitors (i.e. AC-484, K-38) show antitumor activity in melanoma models, PROTACs directed against these regulators of immune system could be investigated in mouse melanoma models in order to improve the response to immunotherapy [264].

As we underlined above, melanoma patients frequently do not respond to target- or immuno- therapies or become resistant to them. Among the biological characteristics responsible of evasion from these treatments, we must consider the Macrophage migration Inhibitory Factor (MIF), a cytokine expressed across tumor and antigen-presenting immune cells, and whose expression is correlated with poor prognosis [265]*.* By using mouse models, a recent paper by Samuels and colleagues evidenced MIF as an oncogenic driver in melanoma with low intratumor heterogeneity, able to induce an immune suppressive TME. The authors validated this finding in melanoma patients being MIF overexpressed in the subgroup with poor survival [266]. Together with findings demonstrating that MIF with its receptor CD74, acts as a pathway involved in melanoma pathogenesis [267, 268]*,* this study supports the use of inhibitors of this pathway to improve melanoma therapy, especially for melanoma with high levels of MIF. The ability of MD13 PROTAC, specifically degrading MIF, to affect melanoma growth and composition/activity of immune cells in TME, should be evaluated in future studies as well as its ability to affect CAR T-cell activation and cytotoxicity against melanoma models, as reported for neuroblastoma models [269].

In the last 5 years several PROTACs have been designed to degrade IDO1, an immunosuppressive protein blocking antitumor immunity via both tryptophan metabolism and nonenzymatic functions (NU223612, NU227326, **2c**). IDO1 is expressed in cells from the TME and in many human cancers, including melanoma, where it is associated with poor prognosis and immune escape [270]. IDO1-PROTACs have been reported to induce in vitro and in vivo antitumor activity in cancers from different origins (glioblastoma and pancreatic, ovarian, prostate cancer) not including melanoma [271, 272]. Antitumor efficacy of PROTACs inhibiting both IDO1 and PD-L1 has been reported in non-small cell lung cancer [273]*.* We envisage further studies of IDO1 PROTACs in melanoma models: these studies could address the challenges faced by inhibitors of either IDO1 or tryptophan pathway and their failure in clinical trials [274], as well as the challenge to identify useful biomarkers able to predict responder patients.

We can also count PROTACs among factors able to improve the safety of CAR-T cell therapy or to regulate activity and abundance of CAR-T [275, 276], as well as the efficacy of Adoptive Cellular Therapy (ACT) using tumor-infiltrating lymphocytes and antigen presenting cells [277]*.* Both these approaches represent promising strategies for metastatic melanoma treatment [278]*.* A Phase I multicenter clinical trial (NCT05107739 [3]), without available results, has been conducted to evaluate safety, tolerability, and preliminary antitumor activity of DeTIL-0255 (drug-enhanced TIL), an autologous ACT originated from a patient’s tumor and expanded ex vivo with NX-0255, a targeted degrader of Casitas B-lineage lymphoma proto-oncogene B , expressed in T cells and regulating immune cell activation [279]*.*

## Conclusion

In the last years enhanced interest has arisen around TPD, and in particular around PROTACs, for their ability to induce antitumor activity and/or antitumor immunity through degradation of proteins involved in melanoma progression or immune cell behaviour. Such PROTACs could potentially improve the outcomes of melanoma patients by affecting tumor cells, those within the TME or both.

Our analysis evidenced the antitumor relevance of PROTACs in in vitro (2D, 3D) and in vivo (mice) melanoma models. In particular, we identified a list of proteins relevant for melanoma pathobiology and response to therapy for which PROTACs antitumor activity has been reported in melanoma preclinical models (Tables 2, 3). Cytoplasmic, ribosomal, nuclear and membrane proteins as well as transcription factors, chromatin modulators and epigenetic readers have been identified among these proteins. Even if at different extend, all proteins are detected in melanoma specimens ranging their expression levels from 20 to 100% (Fig. 7). The expression of most transcripts (BAF, CD147, CDK2, CDK4, MAGE-A3, MITF and TYR) was higher in melanoma specimens compared to normal ones, while NR4A1 was more expressed in normal specimens. Even if not significant, a trend of increment in melanoma (Bcl-xL, CDK6, CREB, MDM2, MEK1/2, NAMPT, PD-L1) or in normal (RPL15, and BRAF, BRD4, CDK12 and NR4A1) specimens was evidenced for several transcripts (Fig. 8). When analysed with the DepMap program, 12 genes have been found essential for 90–100% melanoma cell analysed, 3 genes for 60–75%, while only 3 genes for 0–12%. The list we identified includes proteins that are targeted by drugs currently in the clinical practice for melanoma patients (mutated BRAF and MEK1/2), proteins degraded by PROTACs that entered clinical trials for malignant diseases (Bcl-xL/DT2216 and BRAF V600E/CFT1946, BRD4/RNK05047), proteins whose inhibitors advanced to clinical trials (MAGE-3/GSK 2132231A, MDM2/Alrizomadlin, CD147/metuximab). We are aware that some PROTACs are more promising than other: BRAF (CFT1946), Bcl-xL (DT2216) and BDR4 (RNK05047) degraders entered clinical trials, CDK degraders could be potentially hazardous, transcriptional targets such as MITF could be technically challenging to deploy. Moreover, the relevance of PROTACs that need to be tested in melanoma models, is only “speculative”.

Unfortunately, no clinical trials have been conducted specifically in melanoma, which is included in trials considering refractory or relapsed “solid tumors". To the best of our knowledge, only few clinical trials, recruiting or terminated with not yet available results, have considered melanoma patients among solid tumors for the study of PROTACs (Table 1: PROTACs in clinical trials**)**.

Among the key molecular and cellular mechanisms of resistance to target therapy currently identified in melanoma there are reactivation of the MAPK Pathway, through the production of BRAF splice variants, BRAF amplification, emergence of new mutations in NRAS, or activation of “bypass” pathways, such as PI3K/AKT/mTOR pathway and increased expression of receptor tyrosine kinases. In this view, the use of PROTACs may represent an effective strategy against the raising of new mutations in or increased expression of target proteins. PROTACs only require transient binding to initiate degradation; therefore, "weak" or "promiscuous" binders can be used in their design while still ensuring the recognition of mutated pockets of the target to trigger total protein removal. Moreover, since one PROTACs molecule can degrade many proteins catalytically, they are significantly more effective at handling high protein loads than inhibitors, which require a 1:1 stoichiometry. Regarding the resistance to immunecheckpoint inhibitors, PROTACs strategy could be useful to degrade proteins that suppress the immune system (e.g., STAT3, SHP2, or IDO1) which are difficult to target with traditional small molecules, or to enhance antigen presentation through the increment of the pool of intracellular peptides loaded onto major histocompatibility complex-I derived from protein degradation via the ubiquitin–proteasome system, or to directly degrade checkpoint like PD-L1, acting against the intracellular localized protein, rather than the membrane one, as in the case of the blocking antibody.

To apply a melanoma treatment strategy that exploits the action of PROTACs, it is necessary to take into consideration the possible mechanisms that cells could use to escape the treatment. PROTACs work by recruiting an E3 ligase to the target protein, thus its expression level could be responsible of the efficacy of the PROTACs themselves. However, according to our experience, melanoma cells broadly express high level of VHL protein [199]. Moreover, melanoma cells after prolonged exposure to PROTACs could undergo epigenetic silencing or genetic loss of an E3 ligase, leading to "acquired resistance”. Other possible mechanisms of acquired resistance in a condition of stressed or blocked UPS, forced to work by PROTACs, could be the activation of autophagy to compensate, favoring a compensatory survival process, or the upregulation of proteasome subunits to handle the high flux of ubiquitinated proteins, or the upregulation of DeUBiquitinating enzymes (DUBs).

We strongly expect that understanding the intricacies of PROTACs could allow the definition of therapeutic strategies based on PROTAC technology and could bring significant novelty on melanoma and, more in general, could represent a bridge for cancer treatment (Fig. 4).

The application of PROTACs still faces important challenges, including systemic toxicity and off-tumor adverse effects, due to the selectivity between normal and cancer cells, the reduced/inadequate membrane permeability related to their molecular weight (700–1200 Da in most cases), limited skin/brain penetration, susceptibility to fast degradation by enzymes. To overcome these limitations, “smart/conditional PROTACs” have been developed to control protein degradation in a spatiotemporal or light-dependent manner, and macrocyclization has been proposed to enhance the degradation potency of PROTACs, as well as the selectivity between homologous targets [129]*.*

We are aware that in depth studies are necessary to broaden the range of targetable proteins as well as for the analysis of variables affecting PROTACs efficacy, in terms of selectivity, oral bioavailability, membrane permeability, water solubility, residence time, plasma stability, half-life, clearance and degradation efficiency. New strategies to improve physiochemical performance, as well as to selectively degrade proteins expressed in tumor cells but sparing normal cells could allow low-doses administration and reduced off-tissue effects. Overcoming all these challenges represents a critical priority. Further studies are also needed to overcome the formation of inefficient binary complexes with the E3 ligase or the target protein (Hook effect) frequently formed by high concentrations of PROTACs and responsible of off-target effects [9]*.* Covalent instead of reversible binders, of the E3 ubiquitin ligase, could also increase the degradation efficiencies [61, 67].

Optimization of PROTACs delivery also includes the overcoming of side effects due to the excessive on-target degradation. To improve PROTACs properties, we also encourage further studies addressing these limitations through the development of new strategies such as conjugation of PROTACs with antineoplastic drugs as reported for EGFR-directed PROTAC (nanoassembly strategy) [280]*.* Co-crystal structure analysis and surface plasmon resonance approaches could help for the definition of kinetic parameters driving PROTACs efficacy [281]*.*

Computer- and artificial intelligence- guided PROTACs design could contribute to PROTACs optimization through the application of computational tools such as molecular generation, molecular dynamics simulation and structural prediction, and consequent reduced cost and timeline, and accelerated development.

As cell line dependent degradation has been hypothesized, the development of PROTACs also require analysis in large panels of melanoma cells with different mutational status and different immune cells [282].

Several PROTACs still require validation in animal models and consequently, in clinical trials: in addition to in vitro studies, we consider mice models instrumental in unravelling the potential of PROTACs for melanoma therapy. Small-animal PET imaging could be used to overcome the limitation of knockout mouse models and to define the impact of protein degradation in tumor cells and those belonging to the TME*.* Also the limitation of using the B16F10 mouse melanoma model for in vivo studies, should be overcome by validation of findings in murine melanoma cells harboring the most common mutations found in human melanoma. In fact, even if B16F10 cells recapitulate an intact immune system, its mutation (Ink4a/Arf deletion) partially reflects the human melanoma mutations being *wild type* for both BRAF and NRAS. Also genetically modified mouse models or humanized mice carrying patient-derived xenografts could represent a powerful alternative to syngeneic mouse tumor models to analyze PROTACs antitumor activity.

For a better delivering of PROTACs we also expect the identification of new delivery vectors or the optimization of those already available. Even if several physicochemical property determinants can increase oral absorption for PROTACs, a recent study demonstrating the efficacy of orally administered brain-penetrant PROTACs (CFT1946) in melanoma models, gives us hope for the possible clinical use of PROTACs through this routes [93, 283]. Also intraperitoneally injected covalent Pep-TACs (v9x) demonstrated their ability to cross the blood–brain barrier and to induce antitumor effect and anti-immune response in orthotopic brain tumors, as well as in PD-resistant melanoma [67]. Thuspaves the way for the specific treatment of brain metastases, which are common in advanced melanoma. Subcutaneous or intratumoral treatment should be also considered to increase PROTAC efficacy or to decrease PROTAC toxicity to healthy tissues and organs. To this purpose, gelatin-based hydrogel formulations suitable for these two routes of administration have been developed for target delivery of PROTACs [284]*.* Also topical administration could represent a possible approach to treat melanoma at early stages as reported for topical formulation including Navitoclax. This formulation showed high penetration into the skin, safety and antitumor efficacy in melanoma mouse models [285]. To support this hypothesis, two topically administered PROTACs (AH-001 and GT20029) have accessed phase I and phase II clinical trials for non-malignant diseases (NCT06927960, NCT05428449, NCT06468579, NCT06692465 [3]) [286, 287].

It should be also considered that several PROTACs could be used as a starting point for the identification/development of new degraders for cancer therapy with improved properties. They could belong to the next generation of precision oncology drugs, with higher and more durable effect of already available inhibitors.

We envisage that together with PROTACs, other TPD such as MG could play a pivotal role in next-generation therapeutics. Several Phase I/II clinical trials are ongoing in patients with advanced solid tumors to investigate the safety, tolerability and pharmacokinetics of MGs such as REC-1245, degrading the RNA binding motif protein 39 (RBM39) (NCT06678659, Phase I/II [3]) and MRT-2359 degrading GSPT1 (NCT05546268 [3]). In addition to patients with advanced solid tumors with gene alterations in the RAS-MAPK pathway for whom there are no further treatment options, the two studies also include NRAS mutated melanoma patients.

**Fig. 9:**
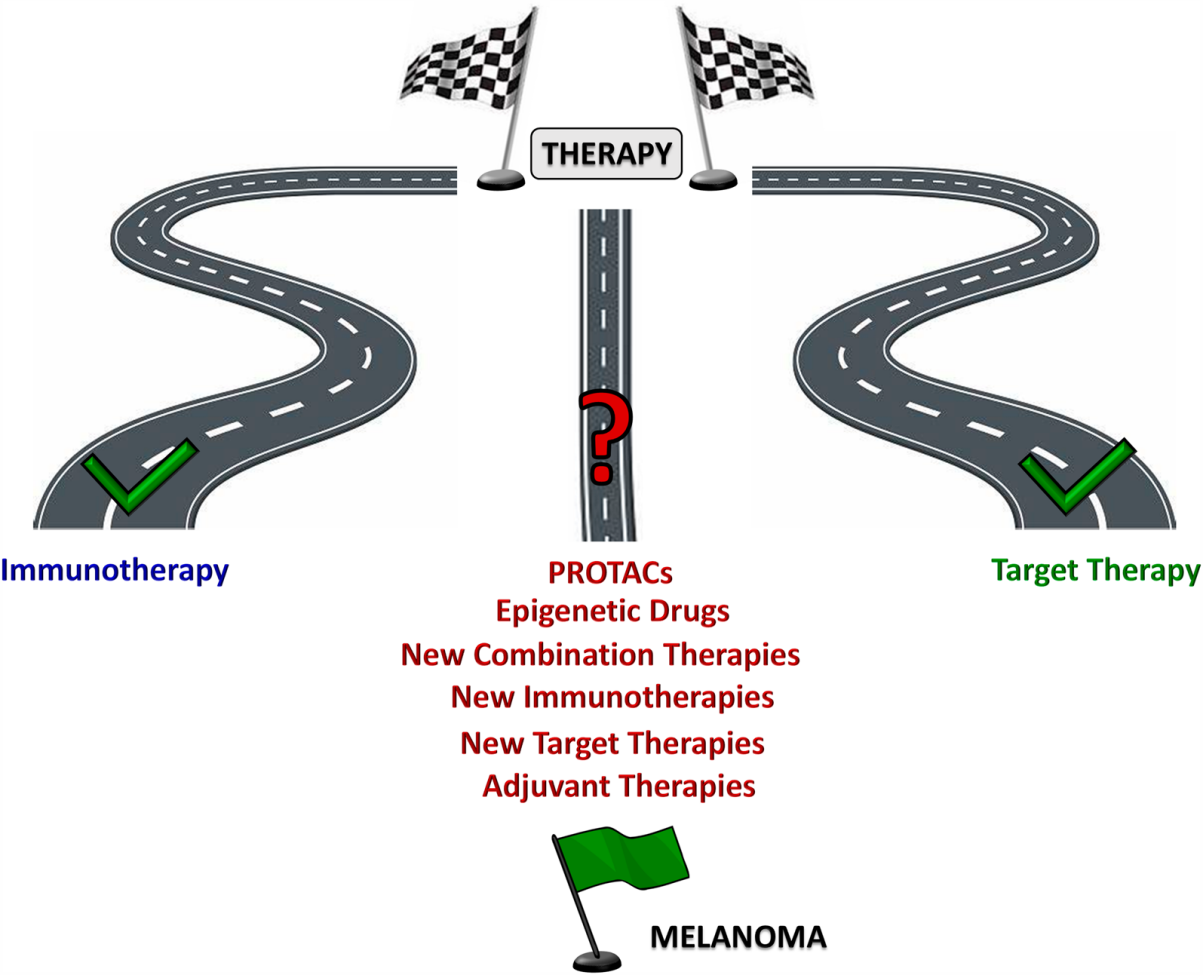
Encouraging preclinical findings, including those with PROTACs, could implement the agent’s armamentarium for melanoma therapy

In conclusion, even though most PROTACs described in this manuscript are at their infancy and there is room for improvement, we strongly believe that PROTACs optimization could represent a possible route for melanoma therapy, especially for patients who do not respond to targeted therapy and immunotherapy, for those who develop resistance following standard treatments or for BRAF *wild type* patients who can benefit only from immunotherapy (Fig. 9).

Harnessing the power of PROTACs holds significant promise for effective clinical outcomes and melanoma treatment, not only affecting tumor cells, but also TME and, in particular, tumor immune cells [241, 288]*.* Combinatorial treatments represent an attractive option: PROTACs integration with target therapy or immunotherapy currently available, should be further explored and could open new avenues for synergistic antitumor effects, through the identification of new synthetic lethality relationship with specific PROTACs.

We envisage PROTACs to represent a useful opportunity not only for the treatment of patients advanced melanoma, but also for cancer types relying on specific proteins for their progression and survival. However, the development of PROTACs is strictly dependent on further studies to deeper understand their lineage-specific role by using cancer cells from different origins and different immune cells [282].

As the expression of POI varies among patients with the same tumor type, clinical trials should consider the selection of patients based on the expression levels of POI.

## Data Availability

Not applicable.

## Abbreviations

ACT: Adoptive Cellular Therapy
R: Arginine
D: Aspartic acid
ATTEC: AuTophagosome TEthering Compounds
AUTAC: AUtophagy-TArgeting Chimera
AUTOTAC: AUTOphagy-TArgeting Chimera
BAF: BRG1-Associated Factor
BET: Bromodomain and Extra-Terminal domain
BRD: BRomoDomain-containing
CREB: CAMP Response Element-Binding protein
cIAP: Cellular Inhibitor of Apoptosis Protein
CRBN: Cereblon
CAR-T: Chimeric Antigen Receptor T cell
CD: Cluster of Differentiation
CDKs: Cyclin-Dependent Kinases
KineTAC: CytoKine receptor-TArgeting Chimeras
CTLA4: Cytotoxic T-Lymphocyte Antigen 4
DDB1: Damaged DNA-Binding protein-1
DAC: Degrader Antibody Conjugates
DAMP: Damage Associated Molecular Patterns
DUB: DeUBiquitinating enzyme
EGFR: Epidermal Growth Factor Receptor
EMA: European Medicines Agency
FOLR1: FOLate Receptor 1
FDA: Food and Drug Administration
GSPT1: G1 to S Phase Transition 1
E: Glutamic acid
G: Glycine
HDAC: Histone DeACetylases
HSP90: Heat Shock Protein 90
HER2: Human Epidermal growth factor Receptor 2
CLIPTACs: In-cell CLIck-formed Proteolysis TArgeting Chimeras
IDO1: Indoleamine 2,3-DiOxygenase 1
IFN-gamma: InterFeroN-gamma
IBG: Intramolecular Bivalent Glue
LAG3: Lymphocyte-Activation Gene 3
K: Lysine
LYTACs: LYsosome TArgeting Chimera
MIF: Macrophage migration Inhibitory Factor
M6PR: Mannose 6-Phosphate Receptor
mTOR: Mechanistic Target Of Rapamycin
MITF: Melanocyte Inducing Transcription Factor
MAGE-A3: Melanoma-Associated antiGEn 3
M: Methionine
MAPK: Mitogen-Activated Protein Kinases
MGs: Molecular Glues
MultiTAC: Multivalent TArgeting Chimera
MDM2: Murine Double Minute 2 homolog
NK: Natural Killer
NF1: NeuroFibromatosis type 1
NAD: Nicotinamide Adenine Dinucleotide
NAMPT: Nicotinamide Phosphoribosyl Transferase
NA: Nicotinic Acid
NR4A1: Nuclear Receptor 4A1
OMV: Outer Membrane Vesicles
PepTAC: Peptide based platform
PTEN: Phosphatase and TENsin homolog
PI3K: PhosphoInositide 3-Kinase
NAPRT: NicotinAte PhosphoRibosyl Transferase
pCREB: Phosphorylated CREB
PHOTAC: PHOtochemically TArgeting Chimera
PDTAC: PhotoDegradation-TArgeting Chimera
PEG: PolyEthylene Glycol
PD-1: Programmed Death protein 1
PD-L1: Programmed Death Ligand-1
AKT: Protein Kinase B
POI: Protein Of Interest
PTP1B: Protein Tyrosine Phosphatase 1B
PROTAC: PROteolysis TArgeting Chimera
ROR1: Receptor tyrosine kinase-like Orphan Receptor 1
RPL15: Ribosomal Protein L15
BRAF: Serine/threonine-protein kinase B-Raf
SIRT2: Sirtuin 2
SM-CMAD: Split-and-Mix Chaperone-Mediated Autophagy-based Degraders
SWI/SNF: SWItch/Sucrose Non-Fermentable
TC-PTP: T-Cell Protein Tyrosine Phosphatase
TPD: Targeted Protein Degradation
TCGA: The Cancer Genome Atlas
TME: Tumor MicroEnvironment
TMB: Tumor Mutational Burden
TFRC/TfR: TransFerrin ReCeptor
TransTAC: TRANSferrin receptor TArgeting Chimera
TYR: Tyrosinase
2D: Two-Dimensional
3D: Three-Dimensional
UPS: Ubiquitin–Proteasome System
V: Valine
VHL: Von Hippel-Lindau
WDR5: WD40 Repeat-containing protein 5
